# Brain connectivity alterations in early psychosis: from clinical to neuroimaging staging

**DOI:** 10.1038/s41398-019-0392-y

**Published:** 2019-02-04

**Authors:** Alessandra Griffa, Philipp S. Baumann, Paul Klauser, Emeline Mullier, Martine Cleusix, Raoul Jenni, Martijn P. van den Heuvel, Kim Q. Do, Philippe Conus, Patric Hagmann

**Affiliations:** 1 Department of Radiology, Lausanne University Hospital (CHUV) and University of Lausanne (UNIL), Lausanne, Switzerland; 2 Dutch Connectome Lab, Department of Complex Trait Genetics, Center for Neurogenomics and Cognitive Research, Amsterdam Neuroscience, VU University, Amsterdam, The Netherlands; 3 Service of General Psychiatry and Center for Psychiatric Neuroscience, Department of Psychiatry, Lausanne University Hospital (CHUV), Lausanne, Switzerland; 4 Center for Psychiatric Neuroscience, Department of Psychiatry, Lausanne University Hospital (CHUV), Lausanne, Switzerland

## Abstract

Early in the course of psychosis, alterations in brain connectivity accompany the emergence of psychiatric symptoms and cognitive impairments, including processing speed. The clinical-staging model is a refined form of diagnosis that places the patient along a continuum of illness conditions, which allows stage-specific interventions with the potential of improving patient care and outcome. This cross-sectional study investigates brain connectivity features that characterize the clinical stages following a first psychotic episode. Structural brain networks were derived from diffusion-weighted MRI for 71 early-psychosis patients and 76 healthy controls. Patients were classified into stage II (first-episode), IIIa (incomplete remission), IIIb (one relapse), and IIIc (two or more relapses), according to the course of the illness until the time of scanning. Brain connectivity measures and diffusion parameters (fractional anisotropy, apparent diffusion coefficient) were investigated using general linear models and sparse linear discriminant analysis (sLDA), studying distinct subgroups of patients who were at specific stages of early psychosis. We found that brain connectivity impairments were more severe in clinical stages following the first-psychosis episode (stages IIIa, IIIb, IIIc) than in first-episode psychosis (stage II) patients. These alterations were spatially diffuse but converged on a set of vulnerable regions, whose inter-connectivity selectively correlated with processing speed in patients and controls. The sLDA suggested that relapsing-remitting (stages IIIb, IIIc) and non-remitting (stage IIIa) patients are characterized by distinct dysconnectivity profiles. Our results indicate that neuroimaging markers of brain dysconnectivity in early psychosis may reflect the heterogeneity of the illness and provide a connectomics signature of the clinical-staging model.

## Introduction

Psychotic disorders, including schizophrenia, are characterized by heterogeneity in terms of etiopathology, clinical presentation and outcome^1^. Although the outcome after a first episode of psychosis is better than traditionally thought^2^, relapses within 5 years from the initial episode are up to 80%^3^. Current definitions of psychotic illnesses are mainly based on relatively short-term evaluations and only poorly consider the long-term, potentially progressive evolution of the illness. Clinical staging, which has proven useful in somatic medicine, is an alternative approach, which captures the degree of disease progression in a given patient and places the person along the continuum of the course of illness^1,4^. Introduced to psychiatry by Fava and Kellner^5^, clinical staging was developed and applied to psychotic disorders by McGorry and collegues^6–8^. This approach allows to distinguish earlier and more benign states from more chronic states, with the aim of selecting the right treatment according to the clinical stage. As a framework, it has proven to be useful in the implementation of early intervention in psychosis by distinguishing an ultra-high risk phase from first-episode and chronic phases. Mapping neurobiological markers, such as brain imaging features, onto clinical stages could further allow us to refine the model and validate the boundaries of the clinical groups, thus broadening our understanding of psychotic disorder pathophysiology^9,10^.

Abnormalities in white matter (WM) and structural brain connectivity are well documented in schizophrenia and have been related to the expression of clinical symptoms, cognitive deficits and differential functional outcome^11,12^. For instance, WM microstructural properties assessed by diffusion-weighted imaging (dMRI) are important in explaining deficits in the processing speed^13–15^, which is one of the most impaired cognitive dimensions in first-episode psychosis and established illness^16,17^ and predicts the functional outcome in patients^18^.

WM alterations are widespread in schizophrenia and affect most of the cerebral lobes in chronic patients^19–21^. From a whole-brain connectivity (or connectome) perspective, patients present a less efficient brain-network organization and a decentralization of core brain regions and hubs^12,21–24^, which are important in maintaining proper information integration underlying cognitive functions in the brain^25,26^. Individuals in the early stages of psychosis appear to have less consistent WM (as well as grey matter (GM)) changes than chronic patients. This observation has led to the hypothesis that there might be a progression or a differentiation of brain connectivity impairments across stages and over the course of the illness from prodromal symptoms to the first-episode of psychosis and, finally, to the relapsing and chronic phases^10,27–30^. Not all patients necessarily progress from one stage to another, which adds complexity to the model. Only a few studies have looked at the early phases after a first episode of psychosis^31–34^, or compared a first episode with multiple episodes^27,35^ or with non-remitting patients. Indeed, there is a 5-year period following the first psychotic episode, called the ‘critical period' by Birchwood and colleagues^36^, during which the most severe brain changes appear to occur^10^.

The objective of the present work is to investigate brain connectomes^37,38^ of early psychosis patients classified in different stages (i.e., stage II, IIIa, IIIb, and IIIc) according to their clinical profiles. Given the tight link between the WM characteristics and the processing speed, we also tested the relationship between the network properties and the processing speed. Our cross-sectional analyses are a first step toward a neuroimaging investigation and validation of the clinical staging model for psychosis illness.

## Materials and methods

### Subjects

A total of 147 subjects (71 early-psychosis patients (EPPs) and 76 healthy controls (HCs)) were included in this cross-sectional study (Table 1). The 71 EPPs (49 males, 26.0 ± 6.2yo) were recruited from an early intervention program (Treatment and Early Intervention in Psychosis Program (TIPP)^39^) of the Lausanne University Hospital, Switzerland. The entry criteria into the TIPP were the following: between 18 and 35 years of age; residence in the catchment area; and meeting the threshold criteria for psychosis according to CAARMS psychosis-threshold subscale^40,41^.

**Table 1:** Demographic and clinical characteristics of the investigated cohort

	HC	EPP	II	III	IIIa	IIIb	IIIc	*p*-value	*p*-value	*p*-value
	*n* = 76	*n* = 71	*n* = 25	*n* = 46	*n* = 17	*n* = 17	*n* = 12	HC/EPP	II/III	IIIa/IIIb/IIIc
Age, years	26.8 (6.1)	26.0 (6.2)	23.5 (4.6)	27.3 (6.5)	26.0 (6.3)	26.6 (6.1)	30.2 (7.1)	0.43	0.012*	0.20
Gender, M/F	48/28	49/22	17/8	32/14	12/5	12/5	8/4	0.45	0.89	0.22
Handedness, R/L	66/10	64/7	25/0	39/7	14/3	15/2	10/2	0.53	0.040*	0.015*
Scanner upgrade,	63/13	46/25	15/10	31/15	10/7	12/5	9/3	0.012*	0.53	0.52
Trio/Prisma										
GAF	83 (5)	59 (11)	58 (12)	59 (10)	55 (9)	63 (9)	60 (10)	< 10^–35^*	0.67	0.073
Processing Speed	53 (9)	41 (12)	44 (13)	38 (11)	35 (10)	37 (15)	42 (6)	< 10^–8^*	0.061	0.40
Diagnosis Sz/Sa/bP/Sf/BP/MD/Pd		41 / 11 / 7 / 4 / 4 / 2 / 2	9 / 4 / 5 / 4 / 2 / 1 / 0	32 / 7 / 2 / 0 / 2 / 1 / 2	14 / 1 / 0 / 0 / 0 / 1 / 1	11 / 2 / 2 / 0 / 1 / 0 / 1	7 / 4 / 0 / 0 / 1 / 0 / 0	–	0.020*	0.32
DOI, years	–	3.0 (3.8)	0.8 (1.0)	4.2 (4.3)	1.9 (1.6)	4.0 (3.3)	7.4 (5.9)	–	0.00028*	< 10^–6^*
DUP, days	–	438 (847)	107 (233)	665 (1029)	320 (545)	585 (963)	1282 (1433)	–	0.016*	0.0029*
PANSS positive	–	13 (4)	13 (5)	13 (4)	14 (4)	12 (4)	13 (5)	–	0.98	0.51
PANSS negative	–	15 (6)	15 (6)	15 (6)	17 (7)	13 (4)	16 (5)	–	0.85	0.16
PANSS general	–	33 (9)	35 (11)	32 (8)	35 (9)	30 (7)	30 (8)	–	0.16	0.067
PANSS total	–	61 (17)	63 (19)	60 (15)	66 (16)	55 (14)	58 (14)	–	0.48	0.10
CPZ, mg/day	–	409.2 (254.7), 12 unmd.	420.5 (218.5), 6 unmd.	403.6 (273.1), 6 unmd.	452.9 (324.2), 3 unmd.	367.9 (222.4), 1 unmd.	388.3 (282.8), 2 unmd.	–	0.67	0.90
CMRS drug N/M/O/S	–	46 / 10 / 6 /0	18 / 6 / 1 /0	28 / 4 / 5 /0	13 / 2 / 2 /0	10 / 2 / 1 /0	5 / 0 / 2 /0	–	0.52	0.63
CMRS alcohol N/M/O/S	–	22 / 37 / 1 / 2	7 / 16 / 1 /1	15 / 21 / 0 /1	7 / 9 / 0 / 1	6 / 7 / 0 / 0	2 / 5 / 0 / 0	–	0.50	0.76

Columns 2,3 report group-mean (standard deviation) values for the 76 healthy controls (HCs) and 71 early psychosis patients (EPPs) included in this study. Columns 4,5 detail the characteristics of two sub-groups of the EPPs’ cohort: stage II (first episode psychosis patients) and stage III (more advanced early psychosis stages after the first psychotic event). Columns 6–8 detail the characteristics of a further subdivision of stage III patients: stage IIIa (non-remitting patients after stage II), stage IIIb (relapse of a psychotic episode after stage II), stage IIIc (two or more relapses after stage II). Columns 9–11: p-values for statistical comparisons between HC/EPP, stages II/III, stages IIIa/IIIb/IIIc groups (one-way ANOVA for continuous and interval variables and chi-square test for categorical variables; *p < 0.05).

*Groups:* *HC* (Healthy controls); *EPP* (Early Psychosis Patients) = stages II + III; Stage III = stages IIIa + IIIb + IIIc

Gender: *M* males, *F* females

Handedness: *R* right-handed, *L* left-handed

Diagnosis: *Sz* schizophrenia, *Sa* schizo-affective disorder, *bP* brief psychotic disorder, *Sf* schizophreniform disorder, *BP* bipolar disorder, *MD* major depressive disorder with psychotic features, *Pd* psychotic disorder not otherwise specified

*DOI:* Duration of Illness at the time of the study, defined as the temporal lapse (years) between the crossing of psychosis threshold (according to CAARMS) and the date of MR imaging.

*DUP:* Duration of Untreated Psychosis at the time of the study, defined as the number of days between the psychosis onset and the date of entry in the TIPP program. DUP information was not available for 12 out of 71 patients: values reported in the table refer to available data

*PANSS:* Positive, negative, general and total PANSS scores

*CPZ:* Chlorpromazine equivalent dose (mg/day); unmd. = unmedicated at the time of the study.

*CMRS drug:* Level of cannabis use, ranked as: none (N), mild (M), moderate (O), severe (S) according to CMRS scale. Data was not available for 9 out of 71 EPPs (4 stage-IIIb and 5 stage-IIIc patients)

*CMRS alcohol:* Level of alcohol use, ranked as: none (N), mild (M), moderate (O), severe (S) according to CMRS scale. Data was not available for 9 out of 71 EPPs (4 stage-IIIb and 5 stage-IIIc patients)

A total of 76 age, gender and handedness-matched HCs (48 males, 26.7 ± 6.1yo) were recruited from the same catchment area; they were not affected by any mood, psychotic or substance-use disorder^41^ and had no first-degree relative with a psychotic disorder. A history of neurological disorder, severe head trauma or mental retardation (IQ < 70) were exclusion criteria for all subjects.

All of the participants provided informed written consent for this study, and the procedure was approved by the Ethics Committee of Clinical Research of the Faculty of Biology and Medicine, University of Lausanne, Switzerland.

### Clinical staging

**Fig. 1:**
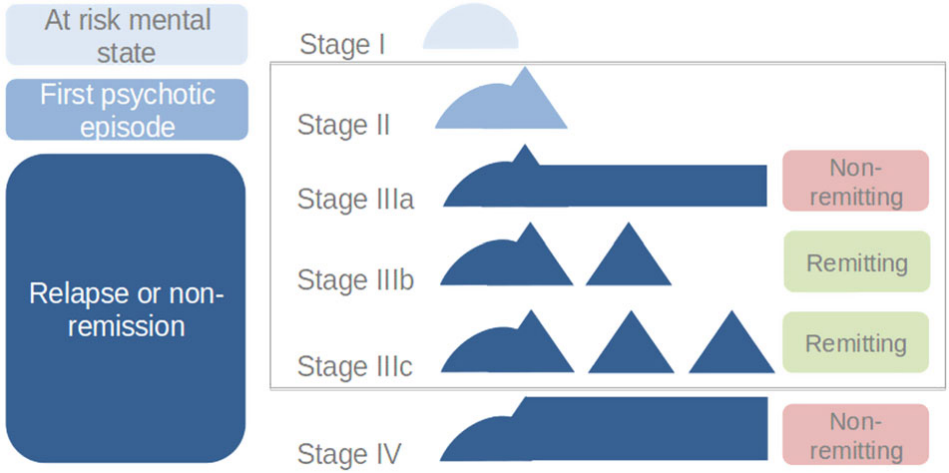
Schematic representation of the clinical staging model (7,41). Stage I: early or late prodromal patients with mild or sub-threshold symptoms; Stage II: first-episode of psychosis (i.e., ‘discrete disorder’); Stage IIIa: incomplete remission; Stage IIIb: one relapse; Stage IIIc: multiple relapses; Stage IV: chronic outcome with severe, persistent illness. This study included patients classified in stages II, IIIa, IIIb or IIIc

The clinical stage was rated as the highest stage achieved at the time of imaging^42^. The patients were stratified into four distinct groups (stages II and IIIa-c (Fig. 1)) based on a consensus assessment by two experienced psychiatrists, according to the clinical-staging model proposed by McGorry and colleagues^7,42^. Any unclear issue was discussed with the case-managers. The subjects in stage II were first-episode psychosis patients, with one psychotic episode according to the CAARMS psychosis-threshold subscale^40^ and no past episodes at the time of the study (i.e., discrete disorder). Patients in stage III were defined as follows: IIIa: incomplete remission from stage II at 12 months after entry to care and following a reasonable course of treatment (>3 months), with duration of illness no longer than 5 years; IIIb: recurrence or relapse of a psychotic episode (i.e., discrete disorder has fully recovered but then relapsed to the full extent described in stage II); IIIc: two or more relapses after stage II with remission between episodes. Further details on the patients’ assessment can be found in SI.1.

### Other clinical and cognitive assessments

In patients, the symptoms severity was assessed with the Positive and Negative Syndrome Scale (PANSS)^43^, and the functioning level was assessed with the Global Assessment of Functioning (GAF) scale^44^. For each patient, a consensus diagnosis^45^ was realized by a senior psychiatrist and a senior psychologist in charge of scale-based assessment over the treatment period, based on DSM-IV criteria^41^. The levels of cannabis and alcohol use were assessed with the Case Manager Rating Scale (CMRS) and ranked as none, mild, moderate, severe or extremely severe^46^. None of the patients had extremely sever level of cannabis or alcohol use. Antipsychotic doses were converted to chlorpromazine equivalents^47,48^. All of the subjects were assessed with the MATRICS Consensus Cognitive Battery^49,50^.

### MRI measurements and connectome reconstruction

Each subject underwent an MR-imaging session on a 3-Tesla Siemens scanner, including magnetization-prepared rapid acquisition gradient echo (MPRAGE) sequence (1-mm in-plane resolution, 1.2-mm slice thickness) and diffusion spectrum imaging (DSI) sequences (257 diffusion-weighted volumes and 1 b0 volume, maximum b-value 8000 s/mm^2^, 2.2 × 2.2 × 3 mm^3^ resolution). During the study, there was a routine MRI-system upgrade from the MAGNETOM-Trio to the MAGNETOM-Prisma Siemens system. Imaging parameters were precisely matched before and after the upgrade, and the same 32-channel head coil was used.

Individual connectomes were estimated by combining MPRAGE and DSI data^51^. Briefly, MPRAGE volumes were segmented into WM, GM and cerebrospinal fluid compartments. The GM was parcellated into 82 (68 cortical and 14 subcortical) regions based on the Desikan-Killiany atlas^52^. Reconstructed DSI-data were used to compute generalized fractional anisotropy (gFA)^53^ and apparent diffusion coefficient (ADC)^23^ scalar maps, and for deterministic streamline tractography. The structural connectivity between each pair of cortical and subcortical regions was quantified as the number of streamlines connecting the two regions, which resulted in 82-nodes, weighted undirected brain-networks. For consistency reasons and to limit possible biases in the network analyses^54,55^, connections that were present in less than 50% of the subjects were discarded.

Further details regarding MRI acquisitions’ parameters, data processing and connectome reconstruction can be found in the SI.2, SI.3.

### Brain connectivity measures

Global and nodal (i.e., specific to single brain regions) connectivity measures were considered. The overall brain network strength was quantified as the total streamline count in the network. The centrality of each brain region in the network was quantified with the nodal strength, which was defined as the weighted sum of the node’s connections. Tract-average gFA^53^ and ADC values were computed for each connection of individual brain networks. gFA and ADC values relate to organizational and microstructural properties of the WM, including myelination levels, axonal packing and fibre coherence^56^. The network efficiency and clustering coefficient^57^ were investigated in supplementary analyses (SI.8, Figure S4−S5).

### Statistical methods

Statistical differences between the subjects’ groups were assessed with multi-factor ANCOVA within a general linear model (GLM) framework. Age, gender, handedness and a ‘scanner-upgrade’ variable were added as co-variates in all of the analyses. Global network analyses were repeated on data acquired on the MAGNETOM-Trio or on the MAGNETOM-Prisma system only to further exclude major effects of the scanner upgrade on the results. The effect size was quantified with the Cohen’s *d* coefficient^58^ between GLM residual distributions, after correcting for covariates. Continuous variables’ cross-group progression was tested with the Jonckheere-Terpstra (JT) test for ordered alternative hypotheses^59^. The false discovery rate correction for multiple comparisons was applied when indicated^60^. Logistic regression was used to assess the cognitive impairments (MATRICS scores) of patients with respect to healthy controls (see SI.4). Relationships between neuroimaging and clinical/cognitive variables were assessed with Pearson’s correlation coefficient (r). A sparse linear discriminant analysis (sLDA)^61,62^ on nodal strength values was performed on patients only to investigate whether distinct brain connectivity features can be associated with distinct clinical stages. Leave-one-out cross validation (LOOCV) error and inter-class distances in the sLDA feature space were used as indicators of classes’ separability. In these analyses, sLDA was meant to explore the neuroimaging patterns characterizing the different early-psychosis stages, and not to generalize to a prediction setting. For further details see SI.5, Figure S1.

## Results

### Subjects and clinical staging

We investigated the brain connectivity alterations that occur in young adults in the early phases of psychosis (EPPs), classified into stages II, IIIa, IIIb and IIIc, and in comparison with healthy controls (HCs). The demographics of the patients and controls, the cognitive and clinical scores, and the related statistical comparisons are reported in Table 1. The patients classified into stage II had significantly shorter duration of illness and duration of untreated psychosis compared to stage III (including, as a whole, stages IIIa, IIIb and IIIc), and there was a significant effect of both duration of illness and duration of untreated psychosis across stages IIIa-IIIb-IIIc. We note that the inter-group differences in duration of illness are implicit to the clinical-staging definition.

In patients, there were no inter-group differences in the GAF, processing speed, PANSS scores, CMRS levels or medication dose (CPZ equivalents) as assessed at the time of MR-imaging. The EPPs had lower GAF and processing speed scores compared to the HCs.

There were no significant differences in the age, gender or handedness between the EPPs and HCs. Stage-II patients were on average younger than stage-III patients, while no age-difference was present among the different sub-groups of the stage-III patients. There was an unbalance in the proportion of right-handed subjects across the patient sub-groups.

### Brain connectivity impairments in stage II and stage III patients

**Fig. 2:**
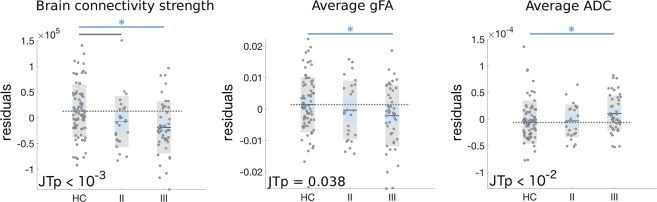
Brain connectivity measures are impaired in stage II and stage III patients. Scatter-plots of overall brain connectivity strength and whole-brain tract-average gFA and ADC for healthy controls (HC), stage II and stage III patients. Residuals after correction for age, gender, handedness and scanner-upgrade are reported. For single-group scatter-plots, the standard error of the mean (light blue area) and the group standard deviation (grey area) around the group mean (black line) are reported. Grey dotted lines indicate the average values of the HC group; blue lines with asterisk represent statistically significant group-differences (uncorrected *p* < 0.05); grey lines represent trend-level differences (uncorrected *p* < 0.1). JT p-values for ordered alternative hypotheses testing ({HC ≥ stage II ≥ stage III} for connectivity strength and gFA, {HC ≤ stage II ≤ stage III} for ADC) are reported

When considering the totality of the EPPs, irrespectively of their classification into clinical stages, we found reduced overall connectivity strength in EPPs compared to HCs (*p* = 0.00086, *d* = 0.55), and trend-level alterations of the whole-brain tract-average gFA (*p* = 0.051, *d* = 0.32) and ADC (*p* = 0.068, *d* = 0.30) values (Figure S2). Using JT-analysis for ordered alternative hypotheses, we found a significant progressive decrease in the brain network connectivity strength (JTp = 0.00027) and average gFA (JTp = 0.038), with connectivity measures higher in HCs, intermediate in stage-II patients and lower in stage-III patients. The tract-average ADC progressively increased across groups (JTp = 0.0070). Post-hoc ANCOVA revealed a significant impairment in the connectivity measures in stage-III patients compared to HCs (overall connectivity strength: *p* = 0.00085, *d* = 0.63; average gFA: *p* = 0.038, *d* = 0.39; average ADC: *p* = 0.021, *d* = −0.42) (Fig. 2). There was a trend of decreased connectivity strength in stage-II patients compared to HCs (*p* = 0.074, *d* = 0.41). No significant pair-wise differences were found between stage-II patients and HCs for the other connectivity measures or between stage-II and stage-III patients. Consistent results were found when analyzing only data acquired before or after the MRI scanner upgrade, or when including right-handed subjects only (for related analyses, see SI.6, SI.7 Table S1-S2, Figure S3). For supplementary analyses with additional network measures, see SI.8 and Figure S4−S5.

### Identification of vulnerable brain regions

**Fig. 3:**
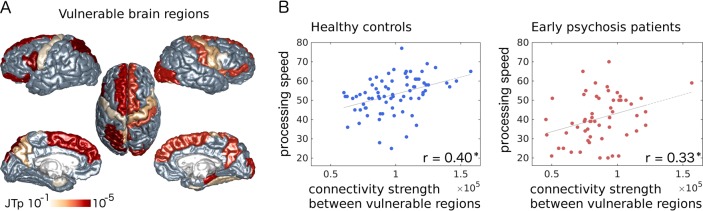
Connectivity strength between vulnerable brain regions selectively correlates with processing speed. **a** Cortical surface plot of nodal JT p-values for ordered impairment of nodal strength values {HC ≥ stage II ≥ stage III} (uncorrected *p* < 0.05; cortical regions with uncorrected *p* ≥ 0.05 are coloured in grey). **b** Relationship between: brain connectivity strength between vulnerable grey matter regions, and processing speed, in HCs (blue dots, *r* = 0.40, *p* = 0.00044) and EPPs (red dots, *r* = 0.33, *p* = 0.012) (*p < 0.05). Grey lines: linear least squares fitting

Considering that the largest whole-brain effect was observed for the overall network strength, we performed a local JT-analysis for ordered nodal connectivity-strength impairments {HC ≥ stage II ≥ stage III} to identify the brain regions that contribute the most to the global effect. A total of 22 out of 82 brain regions demonstrated a significant cross-group progressive decrease in the nodal strength (JT-test, uncorrected *p* < 0.05) (Fig. 3a). Vulnerable regions included the superior frontal gyri, precunei and lateral fronto-basal, somato-motor and temporo-mesial cortices in both hemispheres; the left thalamus, superior-parietal cortex and Heschl’s gyrus; and the right caudate, pallidum and lateral-occipital cortex (Table S3). A total of 2 out of 22 regions (left pars opercularis and left superior-parietal) remained significant after multiple comparison correction (FDR < 0.05). A majority of vulnerable regions ranked among the most central nodes (hubs and rich club) of the brain network (SI.9, Table S3). A comparable set of vulnerable regions was identified when considering only subjects scanned before the MRI system upgrade, or when considering right-handed subjects (SI.6, SI.7, Figure S6).

### Characterization of stage III patients

**Fig. 4:**
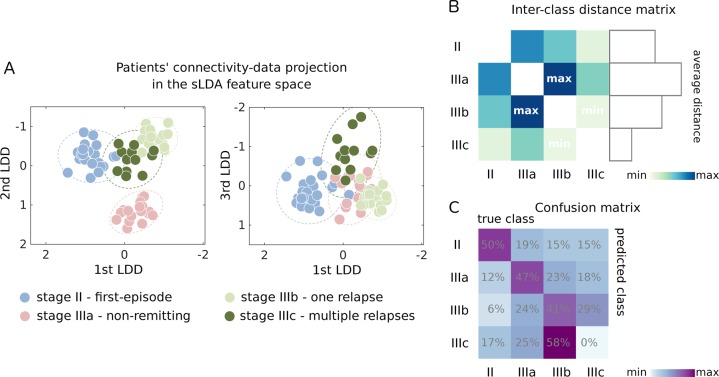
Early psychosis clinical stages are characterized by distinct brain regions’ connectivity profiles. **a** Patients’ representation in the sLDA feature space when sLDA is performed on all the available EPPs. sLDA projects patients’ data (nodal connectivity strength values) onto a three-dimensional feature space where the inter-class separability is maximized. In the plots, each axis represents one of the three linear discriminant directions (LDDs) defining the sLDA feature space, and each point represents a single patient colour-coded according to his/her clinical-staging condition. Dotted lines represent 2-standard deviation intervals for each class. **b** Inter-class Euclidean distance matrix, with distances computed between class centroids in the sLDA feature space. On the right: bar plot representing the average distance of each clinical staging class with respect to the other three groups. **c** Confusion matrix from LOOCV indicating the percentage of subjects (i.e., the number of subjects relative to their true class size) classified in each class. Rows: true classes; columns: LOOCV predicted classes

To explore the heterogeneity of brain connectivity alterations across psychosis stages II/IIIa/IIIb/IIIc, which are possibly associated with different temporal and/or clinical pathways of pathological evolution, we performed an sLDA on the nodal strength values. SLDA is a multivariate technique that identifies directions (linear discriminant directions, LDDs, Figure S7) in the space of the nodal strength values, which maximize the inter-class separation^61,62^. The leave-one-out cross-validation (LOOCV) error of the sLDA classification for stages II/IIIa/IIIb/IIIc was 0.60 (below chance-level LOOCV-error = 0.75 and naïve-classifier LOOCV-error = 0.65, see SI.5 for further details), suggesting that the different clinical stages are characterized by distinct nodal connectivity patterns. Figure 4a shows the spatial organization of the patients in the sLDA feature space. We note that, in the sLDA feature space, there was no obvious pattern in the distribution of the subjects with respect to the scanner upgrade or to the subjects’ handedness, indicating that these two factors are not major drivers of data classification (Figure S8-S9). When considering the inter-class distances in the sLDA feature space, non-remitting patients (stage IIIa) formed the most separable class (Fig. 4b). On the other hand, the remitting patients with multiple relapses (stage IIIc) formed the less separable class, and the minimum inter-class distance was observed between the two remitting groups (stages IIIb and IIIc). When performing the cross-validation procedure, the majority of stage IIIc patients were (mis)classified in the class IIIb (Fig. 4c), suggesting an overlapping dysconnectivity signature between stages IIIb and IIIc.

### Relationship with clinical and cognitive profiles

Among the MATRICS domains, the processing speed was the most impaired in the patients (including all stages) compared to the controls (logistic regression analysis, SI.4). There was a significant positive correlation between the processing speed and the overall network strength in both HCs (*r* = 0.26, *p* = 0.024) and EPPs (*r* = 0.26, *p* = 0.049). This correlation was driven by the connectivity strength between the 22 vulnerable regions (HCs: *r* = 0.40, *p* = 0.00044; EPPs: *r* = 0.33, *p* = 0.012) (Fig. 3b), and no relationship was found between the processing speed and the connectivity strength in the remainder of the network. In patients, we investigated the subjects’ scores along the three discriminant directions (LDDs) identified in the sLDA. The first and third-LDD patients’ scores correlated with the duration of illness (respectively, *r* = −0.30, *p* = 0.00075 and *r* = −0.38, *p* = 0.0011); the first-LDD scores correlated with the processing speed (*r* = 0.28, *p* = 0.037). We did not find any relationship between the connectivity measures (overall network and vulnerable regions connectivity strength, average gFA, average ADC) and the duration of illness, duration of untreated psychosis, PANSS scores, GAF scores, CMRS levels and medication dose in patients.

## Discussion

White-matter alterations that occur in the early stages of psychosis may parallel the course of the illness and differentiate the clinical subtypes^9,10,63^. In this cross-sectional work, we investigated the white-matter connectivity of early-psychosis patients (EPPs) in different clinical stages after the first psychotic episode and in comparison with healthy controls (HCs). We found that the EPPs have reduced brain connectivity strength, lower gFA and higher ADC values compared to the HCs. Such connectivity impairments are more severe in the advanced (early) stages than in the first-episode of psychosis (stage II), and they converge on a set of vulnerable brain regions whose connectivity strength selectively correlates with the processing speed. Finally, using a linear discriminant analysis technique, we showed that clinical subtypes are characterized by distinct brain-connectivity profiles.

Our results on brain connectivity alterations across early-psychosis stages complement recent studies that indicate progressive brain tissue atrophy across clinical stages^10,26^, and progressive dysconnectivity from recent-onset to chronic schizophrenia patients^32,64^. While our data, being cross-sectional, cannot directly confirm a progressive change of WM-connectivity over the early course of the pathology, they reveal an association between WM-connectivity alterations and clinical stages. These alterations are spatially diffuse in the brain, but they converge on a vulnerable subnetwork that spans frontal, inter-hemispheric, cortico-thalamic and striatal circuits. This vulnerable subnetwork spatially aligns with the literature on WM-impairments in schizophrenia^12,21,22,65^ and partially overlaps (~40%) the brain’s ‘affected-core’ that we detected in previous work on chronic patients^23^. Within the subnetwork, the most significant effects were found in the left hemisphere, in the superior parietal and frontal cortices (which suggests an involvement of the superior longitudinal fasciculus, previously associated with a high risk for developing psychosis^29^) and in the pars opercularis of the inferior frontal gyrus (a language area that, together with the Heschle’s gyrus, has been related to auditory hallucinations^66–68^). The vulnerable subnetwork includes the main brain-network hubs (namely, the superior frontal and superior parietal cortices, precuneus, insula and thalamus), which are consistently implicated in schizophrenia pathophysiology^21,22^ and whose early impairment might relate to genetic and clinical risk factors^69–71^. Our findings corroborate the dysconnectivity hypothesis of schizophrenia pathophysiology and suggest that the early decentralization of vulnerable brain regions, including hubs, might play a role in the aetiology of the disorder.

The connectivity strength between vulnerable brain regions selectively correlates with the processing speed, in both patients and controls, which indicates an involvement of these regions and connections in maintaining fast response and proper information integration in the brain. The processing speed is a core dimension of the cognitive deficit that is observed not only in chronic schizophrenia^13,14^ but also in first-episode and clinical high-risk populations^17,72,73^, and it could mediate working memory impairments^15^. In our sample, the processing speed was impaired in EPPs compared to HCs, with a statistical effect that exceeded that of other cognitive domains. Our results suggest that inter-subject WM variability in connections vulnerable to psychosis could mediate inter-subject variations in the processing speed.

To assess the connectivity strength and microstructural properties of WM tracts in patients and controls, we investigated the number of streamlines (NOS), average gFA and average ADC values. These measures indirectly relate to the tract volume and to tissue microstructural properties such as local axonal packing, membrane permeability and myelin properties^56,74^. Alterations in NOS, gFA and ADC are compatible with neurobiological processes that have been associated with schizophrenia, such as neuroinflammation, myelin impairment and loss of tissue volume^75–77^. It has been suggested that oxidative stress is an important risk factor for developing schizophrenia. Redox dysregulation interacts with neuroinflammation and glutamatergic hypofunction to impair oligodendrocyte maturation and myelination^78–80^. In early psychosis, WM gFA values relate to brain concentrations of glutathione, a fundamental antioxidant that prevents oxidative stress and oligodendrocytes sufferance^78^. Elevated ADC values have been directly associated with demyelination processes in animal models with myelin deficiency^81^, and both gFA and ADC impairments in schizophrenia have been related to deficits in the density or myelination of axonal fibers^82^. In schizophrenia, NOS-connectivity relates to cytoarchitectonic abnormalities of pyramidal neurons, which are centrally implicated in the disorder^83^. In this study, we found that NOS-connectivity bears the largest effect for progressive connectivity changes across psychosis stages. While this result suggests that NOS might be sensitive to pathological processes that occur early and persist over the development of the disease, this measure remains an unspecific marker of WM-organization. Future studies that employ additional dMRI-techniques for WM microstructural assessment^84,85^ could better characterize the neurobiological processes that underlie macro-scale connectivity alterations across psychosis stages.

Although our analyses suggest a progressive WM-connectivity impairment from the first episode to more advanced (early) psychosis stages, we did not find in the first instance direct correlations between neuroimaging markers and duration of illness. A possible explanation for this finding is that connectivity alterations follow spatially non-homogeneous and temporally non-linear patterns over the course of the pathology, with periods of faster and slower WM changes localized in different areas of the brain^32,34^. A second complementary explanation is that different clinical subtypes can experience different spatio-temporal patterns of WM alteration. For example, it is plausible that an early-psychosis patient with a non-remitting course of illness could present a different or more severe dysconnectivity pattern than a relapsing-remitting patient, with duration of illness being equal. A large longitudinal study on first-episode schizophrenia found that individuals with extended periods of relapse had more severe brain-tissue atrophy compared to brief- or no-relapse conditions^86^, while GM-volume alterations measured at the time of the first psychotic episode partially predicted the course of psychosis into continuous or remitting illness^34^. Moreover, WM-changes detected after a first psychotic episode can partially reverse with the remission of acute psychotic symptoms^87^.

To investigate whether different early-psychosis clinical subtypes are associated with distinct WM-connectivity patterns, we performed an exploratory sparse linear discriminant analysis (sLDA) on nodal connectivity-strength values. Our analysis suggests that distinct brain connectivity features characterize the clinical stages and identifies axes of pathological evolution in terms of both (cross-sectional) temporal progression (qualitatively, 1^st^ and 3^rd^ LDDs (Fig. 4a), with patients’ scores along these dimensions correlating with the duration of illness) and clinical differentiation in terms of relapsing-remitting or non-remitting pathology (2^nd^ LDD, Fig. 4a).

It should be noted that additional factors that were not accounted for in our analyses, such as diagnostic heterogeneity, number of relapses and differential longitudinal follow-up, could blur the boundaries of the clinical groups (stages II, IIIa, IIIb, and IIIc) and the corresponding neuroimaging markers. Our data are cross-sectional and it is not possible to foresee the pathological evolution of single individuals. In addition, our sample-size is relatively small, which limits the statistical power and the generalizability of our exploratory sLDA analysis to a classification or predictive setting. Finally, although no relationship was found between brain-connectivity measures and antipsychotic dose, and no differences in CPZ equivalents were found between patient subgroups, possible iatrogenic or other secondary effects on the reported neuroimaging measures cannot be completely excluded. While some studies have shown that medication can negatively impact WM connectivity^88,89^, others found no effect^90^, and this aspect should be further assessed in future research.

In conclusion, our work shows that neuroimaging markers of brain dysconnectivity and core-network decentralization are more severe in more advanced early-psychosis stages and typify relapsing-remitting and non-remitting clinical profiles, thus providing a connectomics perspective of the clinical-staging model. Based on these results, we expect that the investigation of large datasets (including stage-I and stage-IV individuals), the inclusion of additional clinical, neurobiological and multimodal brain-connectivity features, and longitudinal designs combined with machine learning approaches will significantly contribute to model and understand the unfolding of psychosis-spectrum disorders across clinical stages.

## Supplementary information

Supplemental Information
