# Supplementary material for: Brain connectivity alterations in early psychosis: from clinical to neuroimaging staging
Source: Transl Psychiatry. 2019 Feb 4;9:62. doi: 10.1038/s41398-019-0392-y (PMC6362225; doi:10.1038/s41398-019-0392-y)
Supplement: Supplementary file 1 — Supplemental Information [file 41398_2019_392_MOESM1_ESM.docx]

**Brain connectivity alterations in early psychosis: From clinical to neuroimaging staging**

Alessandra Griffa, PhD^a b^ *; Philipp S. Baumann, MD^c d^ *; Paul Klauser, MD-PhD^c d^; Emeline Mullier, Msc^a^; Martine Cleusix, MSc^d^; Raoul Jenni, PhD^d^ ; Martijn P. van den Heuvel, PhD^b^; Kim Q. Do, PhD^d^; Philippe Conus, MD^c#^; Patric Hagmann, MD-PhD^a#^

a *Department of Radiology, Lausanne University Hospital (CHUV) and University of Lausanne (UNIL), Lausanne, Switzerland*

b *Dutch Connectome Lab, Department of Complex Trait Genetics, Center for Neurogenomics and Cognitive Research, Amsterdam Neuroscience, VU University, Amsterdam, The Netherlands*

c *Service of General Psychiatry and Center for Psychiatric Neuroscience, Department of Psychiatry, Lausanne University Hospital (CHUV), Lausanne, Switzerland*

d *Center for Psychiatric Neuroscience, Department of Psychiatry, Lausanne University Hospital (CHUV), Lausanne, Switzerland*

* A. Griffa and P.S. Baumann contributed equally as first authors

# P. Hagmann and P. Conus contributed equally as senior authors

**Online Supplementary Information**

**1. Inclusion criteria and clinical assessment of early psychosis patients**

**2. MRI acquisition reporting**

**3.** **MRI processing, connectome reconstruction and network measures**

**4. Logistic regression analysis of MATRICS items**

**5. Sparse Linear Discriminant Analysis**

**6. Brain connectivity analyses restricted to MAGNETOM-Trio or MAGNETOM-Prisma data**

**7. Brain connectivity analyses restricted to right-handed subjects**

**8. Network measure impairments in early psychosis patients**

**9. Brain Network Hubs**

**References**

**Supplementary Figures and Tables:**

**Figure S1 – sLDA training and LOOCV errors**

**Figure S2 – EPP-HC group-comparison**

**Figure S3 – HC, stage-II and stage-III group-comparison for Trio and Prisma cohorts**

**Figure S4 – EPP-HC network efficiency and clustering coefficient**

**Figure S5 – Progressive impairment of network efficiency and clustering coefficient**

**Figure S6 - Vulnerable brain regions identified on the complete and Trio datasets**

**Figure S7 – Linear discriminant directions’ loadings**

**Figure S8 – sLDA feature space and scanner upgrade**

**Figure S9 – sLDA feature space and subjects' handedness**

**Figure S10 – Connectivity strength and processing speed in Trio and Prisma datasets**

**Table S1 – Trio and Prisma cohorts**

**Table S2 – Summary of group-differences for Trio and Prisma cohorts**

**Table S3 – Vulnerable brain regions**

**1. Early psychosis patients' inclusion criteria and clinical assessment**

71 early psychosis patients (EPPs) (49 males, 26.0±6.2yo) were included in this study. Patients were recruited during the 3-year follow-up at the Treatment and Early Intervention in Psychosis Program ('TIPP program'^1^) (66 patients) or Minkowski section (5 patients) at Lausanne University Hospital, Switzerland. The entry criteria into the program were the following: (i) between 18 and 35 years of age; (ii) residence in the catchment area (Lausanne and surroundings); (iii) meeting the threshold criteria for psychosis, as defined by the ‘Psychosis threshold’ subscale of the Comprehensive Assessment of At Risk Mental State (CAARMS)^2^. While MR imaging was proposed to the patients at the beginning of the program, some patients accepted the MR assessment only after a follow-up period.

In an attempt to stratify the patients’ cohort, clinical staging was rated as the highest stage achieved at the time of MR imaging^3^. Clinical information was collected by the case managers as follows: i) A specially designed questionnaire^1,4^ was completed for all patients enrolled in the program by case managers who have up to 100 contacts with patients during the first 3 years of treatment. The questionnaire includes exhaustive patients' characteristics (demographics, medical history, functioning, etc.), it is completed on the basis of information gathered with patients and their family over the first few weeks of treatment and can be updated during the follow-up if new information emerges. ii) Follow-up assessments, exploring various aspects of treatment and co-morbidities as well as evolution of psychopathology and level of functioning, were conducted by a research psychologist and by case managers after 2, 6, 12, 18, 24, 30 and 36 months in treatment. iii) Medical documents and discharge reports were collected at the end of any hospitalization. This information was used in a consensus assessment by two experienced psychiatrists (P.S. Baumann and P. Klauser) to stratify the patients into four distinct groups (stage II, IIIa, IIIb, IIIc), according to the clinical staging model^5,3^.

Diagnosis, symptoms severity, global functioning, duration of illness (DOI) and duration of untreated psychosis (DUP) were assessed for each patient.

A consensus diagnosis procedure was elaborated in the framework of the TIPP program and is described elsewhere^4^. Briefly, the consensus diagnosis is realized by a senior psychiatrist and the senior psychologist who is in charge of scale-based assessment (DSM-IV criteria^6^) of the patient over the treatment period, and it considers the diagnosis reported by the treating psychiatrist in all medical documents and at the end of any hospitalization, and the longitudinal assessments performed by the clinical case managers over the 3 years of treatment.

Level of symptoms was assessed with the Positive and Negative Syndrome Scale (PANSS)^7^, including Positive, Negative, General and Total scores.

Level of functioning was assessed with the Global Assessment of Functioning (GAF) scale^6^.

DOI was defined as the temporal lapse (measured in years) between the crossing of psychosis threshold (according to the CAARMS criteria) and the date of MR imaging.

DUP was defined as the number of days between the psychosis onset and the date of entry in the TIPP programme.

For patients under medication at the time of MR assessment, antipsychotic dose was assessed and converted to chlorpromazine equivalents (CPZ)^8,9^.

**2. MRI acquisition reporting**

Each subject underwent an MRI session on a 3-Tesla Siemens Medical Solutions scanner equipped with a 32-channel head coil, and located at Lausanne University Hospital, Lausanne, Switzerland. The MRI session included a magnetization-prepared rapid acquisition gradient echo (MPRAGE) sequence and a diffusion spectrum imaging (DSI) sequence. During the period of this study there was a routine MRI-system upgrade from MAGNETOM-Trio Tim to MAGNETOM-Prisma Siemens system. MPRAGE and DSI sequences were carefully matched and the same 32-channel head coil was used before and after the upgrade. We report below the complete MRI acquisition details:

MPRAGE - magnetization-prepared rapid acquisition gradient echo sequence

TE (echo time) 2.98 ms

TR (repetition time) 2300 ms

FA (flip angle) 9 deg

TA (acquisition time) 3:47 min

FOV (field of view) 256 x 240 x 192 mm

In-plane matrix size 256 x 240 voxels

Slice thickness 1.2 mm

Interslice gap none

Slice orientation sagittal

3D matrix size 256 x 240 x 160 voxels

Phase encoding direction A >> P

Parallel imaging method GRAPPA (acceleration factor PE 3)

Fat suppression none

Shimming standard mode

Brain coverage whole-brain

DSI – diffusion spectrum imaging sequence

TE (echo time) 144 ms

TR (repetition time) 6800 ms

TA (acquisition time) 14:59 min

Number of gradient directions 128

B-values from 500 to 8'000 s/mm²

Number of b=0 images 1

Number of averages 0

Acquisition scheme cartesian sampling of the q-space, q4 scheme

Cardiac gating no

FOV (field of view) 212 x 212 x 114 mm

In-plane matrix size 96 x 96 voxels

Slice thickness 3 mm

Interslice gap none

Slice orientation transversal

3D matrix size 96 x 96 x 34 voxels

Phase encoding direction A >> P

Parallel imaging method GRAPPA (acceleration factor PE 2)

Fat suppression Fat sat.

Shimming standard mode

Brain coverage whole-brain (cerebellum excluded)

**3. MRI-data processing, connectome reconstruction and network measures**

Individual connectomes were estimated by combining MPRAGE and DSI data.

For each subject, the MPRAGE volume was first segmented into WM, GM and cerebrospinal fluid (CSF) compartments using FreeSurfer software v5.0.0^10^. GM volume was then parcellated into 82 regions of interest (68 cortical and 14 subcortical regions) according to the Desikan-Killiany atlas^11^. The MPRAGE volume was linearly register to the DSI b0-volume using FSL software^12^, and the same geometrical transformation was applied to the WM and GM-parcellation masks to bring them into the native diffusion space.

DSI volumes were visually inspected for possible signal drop-outs over the scanning time. Signal drop-outs can indicate motion artifacts and are indicative of poor data-quality^13^. None of the subjects included in this study presented signal drop-outs warranting exclusion from further analyses. DSI data were reconstructed according to Wedeen and colleagues^14^. Generalized fractional anisotropy (gFA)^15^ and apparent diffusion coefficient (ADC) scalar maps were computed form reconstructed DSI data. Local diffusion orientation information was converted to diffusion peaks (for a maximum of three peaks per voxel) and used as input to a deterministic streamline tractography algorithm with the following parameters: 32 seeds per WM voxel, per diffusion direction; termination criteria: 60-degree angle between following propagation steps or reaching the WM/GM boundary. A weighted, undirected brain connectivity network (or connectome) was reconstructed for each subject by filtering the whole-brain tractogram according to the termination coordinates of the single streamlines and using the Connectome Mapper Toolkit^16^. The structural connectivity strength between each pair of cortical and subcortical regions was quantified as the number of streamlines (NOS) that connected the two regions. For consistency reasons and to limit false-positive biases in the network analyses^17,18^, connections that were present in less than 50% of the subjects (including both healthy controls and patients) were discarded.

For each individual connectome, the following global network measures were computed: whole-brain network strength (*NS*), quantified as the total number of connecting streamlines in the network; network efficiency (*NE*), defined as the harmonic average of the inverse weighted shortest path lengths between pairs of brain regions in the^19^; average clustering coefficient (*NC*), defined as the average clustering coefficient of the single brain regions in the network^19^. The clustering coefficient of a single brain region is defined as the weighted fraction of the region's direct neighbors in the network which are also neighbors of each other’s^20^. The network efficiency and average clustering coefficient of the brain network reflect the overall capacity of functional integration and segregated (specialized) functional processing of the network^22,22^.

The centrality within the overall brain network of each individual brain region *i* was quantified with its nodal strength *Si*, defined as the weighted sum of the node's connections^19^.

**4. Logistic regression analysis of MATRICS items**

All the subjects included in the study were assessed with the MATRICS Consensus Cognitive Battery (MCCB)^23,24^. In order to identify the MATRICS items (or cognitive domains) mostly impaired in early psychosis patients (all stages included) compared to healthy controls, we ran a multivariate logistic regression analysis^25^ with the following MATRICS domains listed as predictor values: (1) processing speed, (2) attention/vigilance, (3) working memory, (4) verbal learning, (5) visual learning, (6) reasoning and problem solving^23,24^.

The standardized regression coefficients (*b_i_*) of the six MATRICS predictors and their relative p-values (p), were as follows: (1) processing speed: *b_1_*=1.1945 (p=0.0054), (2) attention/vigilance *b_2_*=0.0409 (p=0.90), (3) working memory *b_3_*=0.3491 (p=0.31), (4) verbal learning *b_4_*=0.7102 (p=0.040), (5) visual learning *b_5_*=-0.6776 (p=0.040), (6) reasoning and problem solving *b_6_*=0.1270 (p=0.65). These values indicate that, in the investigated sample, the processing speed is the most discriminative predictor between patients and control subjects, which is in agreement with available literature on schizophrenia patients^26,27^. We note that, according to the MCCB, the processing speed was assessed by combining three subtasks, namely Verbal Fluency, Trail Making Test part A and BACS Symbol Coding.

**5. Sparse Linear Discriminant Analysis**

We performed a linear discriminant analysis to investigate whether brain connectivity patterns can differentiate patients in different stages of early psychosis (stages II, IIIa, IIIb and IIIc) and can provide useful information on dysconnectivity features specific to pathological stages. Linear discriminant analysis is a multivariate, supervised feature extraction and classification technique that identifies directions (namely, linear discriminant directions (LDDs)) in the explanatory variables' space which maximize the inter-class separation. The connectivity strength values (*Si*) of the 82 brain regions were used as explanatory variables. Leave-one-out cross validation (LOOCV) and nested-LOOCV were used to assess the inter-class discrimination power. The pair-wise Euclidean distance between the patient groups’ centroids after projecting the data onto the LDDs were used to describe the distribution of the data in the feature space.

Considering the small ratio between the number of data points (71 patients) and the number of explanatory variables (82 *Si* values per subject), we added an elastic-net sparsity penalty to the classic linear discriminant analysis formulation^28^. The elastic net regularization includes two penalty terms on the coefficients of the discriminative functions (or loadings) estimated by the linear discriminant analysis: a l1-norm penalty term and a l2-norm penalty term. The first term yields sparse loading-vector estimates. The second term favors correlated features (i.e., correlated nodal strength values across subjects) to be assigned similar loadings. According to the method and implementation proposed by Clemmensen and colleagues^28^, we set the weight of the l2-norm term equal to the default value 1e-3. We then let vary the parameter λ that tunes the contribution of the l1-norm term to the sparse linear discriminant analysis (sLDA) solution. The value of λ bounds the number of non-zero sLDA loadings per discriminant direction in the final solution. The range of λ values [10, 45] was tested in our analysis, and leave-one-out cross validation error (LOOCV-error) and training error (i.e., the classification error when the complete dataset is used to estimate the LDDs) were computed (FigureS1). For larger λ values (λ>45), the sLDA optimization procedure did not reach convergence. The training error is expected to monotonically decrease for higher value of λ (i.e., when a larger number of nodal strength values are considered for the patients' classification). The LOOCV-error is expected to initially decrease, reach a minimum and then increase for increasing values of λ. The region where the LOOCV-error increases indicates to data over-fitting. The minimum LOOCV-error was obtained for λ=38 (LOOCV-error=0.60, training error=0.02) (FigureS1). sLDA results reported in the main manuscript refer to the solution for λ=38. The LOOCV-error achieved in the sLDA-classification outperforms the chance-level for a 4-class classification problem (chance-level error = 0.75) and the performance of a naïve classifier that classifies all data points into the most populated class (no-information error rate = 0.65).

We note that the usage of cross validation on the same dataset for both the model validation (i.e., hyperparameter (λ) setting) and the model testing (i.e., classification error assessment) operations can lead to an under-estimation of the classification error. Therefore, we additionally performed a nested cross-validation, including an outer LOOCV dataset subdivision for the validation/test partition, and an inner LOOCV dataset subdivision for the training/validation partition. The nested LOOCV procedure selected a regularization weight λ=-38.9±3.5 (average and standard deviation over 71 runs, 71 being the number of patients included in the classification analysis; the λ-value corresponding to the minimum LOOCV-error was selected at each iteration). The LOOCV-error estimated from the nested procedure was equal to 0.62, i.e., inferior to the chance-level and no-information error rates.

Considering the limited sample size available for this study, the sLDA analysis reported in this study has the objective of investigating connectivity differences among multiple and clinically heterogeneous patients’ groups, and is not meant to generalize to a classification or predictive setting.

Individual nodal *Si* values were corrected for the effect of age, gender, handedness and scanner-upgrade before performing the sLDA. The effect of the co-variates was estimated on the healthy controls’ group with stepwise multilinear regression.

All statistical analyses were performed using MATLAB and Statistics Toolbox Release R2016b.

**6. Brain connectivity analyses restricted to MAGNETOM-Trio or MAGNETOM-Prisma data**

Global network analyses were repeated on data acquired only before (MAGNETOM-Trio) or only after (MAGNETOM-Prisma) the MRI scanner upgrade, to exclude major effects of the scanner upgrade on the results. Results of these analyses were coherent with the findings obtained when considering the whole dataset (and when including a 'scanner-upgrade' categorical covariates). We also assessed the distribution of the patients in the sLDA feature space with respect to the scanner upgrade variable. We did not find any spatial patterns in the distribution of subjects imaged before or after the scanner upgrade in the sLDA feature space, indicating that the scanner upgrade is not a major driver of data classification (FigureS8).

The number of subjects included in the different control and clinical sub-groups, when considering MAGNETOM-Trio or MAGNETOM-Prisma subjects only, is reported in TableS1. For a summary of group-comparisons across the different cohorts, see TableS2.

*MAGNETOM-Trio data*

We first compared the global connectivity measures of the early psychosis patients (EPPs) (irrespectively of their classification into clinical stages) with the healthy controls (HCs) scanned before the MRI system upgrade (46 EPPs and 63 HCs). We found reduced overall connectivity strength (p=0.00015), and a trend-level increase of whole-brain tract-average ADC values (p=0.081) in EPPs compared to HCs. Whole-brain tract-average gFA values were on average lower in EPPs than in HCs, but the effect did not reach statistical significance in this sample (p=0.18).

The patients acquired on the MAGNETOM-Trio system were then subdivided into the clinical stages II (n=15) and III (n=31).

Using a JT-analysis for ordered alternative hypotheses, we found a significant progressive decrease of the brain network connectivity strength (JTp=0.000067) and a significant progressive increase of the whole-brain tract-average ADC (JTp=0.011). The p-value for the JT-analysis on the whole-brain tract-average gFA was not significant (JTp=0.11) (FigureS3).

Post-hoc ANCOVA revealed a significant impairment in the connectivity measures in stage-III patients compared to HCs (overall connectivity strength: p=0.00014; average ADC: p=0.049) (TableS2). There was a trend for decreased connectivity strength in stage-II patients compared to HCs (p=0.088). No other pair-wise differences of global network measures were found between the HC, stage-II, and stage-III groups.

Finally, we performed a local JT-analysis for ordered nodal connectivity-strength impairments {HC ≥ stage II ≥ stage III} to identify the brain regions that contribute the most to the reported global effect on brain network connectivity strength. A total of 30 out of 82 brain regions demonstrated a significant cross-group progressive decrease in nodal strength (JT-test, uncorrected p<0.05). 8 of these 30 regions survived multiple comparison correction (FDR<0.05), namely the left lateral orbitofrontal, left pars opercularis (which also survived FDR-correction in the main analyses on Trio and Prisma data), bilateral superior frontal, left superior parietal (which also survived FDR-correction in the main analyses), left inferior parietal, right cuneus and right parahippocampal cortices. In the main analyses on Trio and Prisma data we found 22 vulnerable regions with a decreasing pattern of connectivity strength from HC to stage II to stage III patients (uncorrected p<0.05): 86% of these regions (19 out of 22 regions) overlap the results obtained on Trio data only (FigureS6).

Age, gender and handedness were included as covariates in all the analyses.

*MAGNETOM-Prisma data*

We compared the global connectivity measures of the early psychosis patients (EPPs) (irrespectively of their classification into clinical stages) with the healthy controls (HCs) scanned after the MRI system upgrade (25 EPPs and 13 HCs). There were no statistically significant differences of overall connectivity strength (p=0.84) and average ADC (p=0.67) values in EPPs compared to HC, when including only subjects scanned on the MAGNETOM-Prisma system. We found a trend-level decrease of whole-brain tract-average gFA values in EPPs compared to HCs (p=0.082). An explanation for the absence of statistically significant group-differences in this cohort can be the small size of the HC group.

The patients acquired on the MAGNETOM-Prisma system were then subdivided into the clinical stages II (n=10) and III (n=15).

Using a JT-analysis for ordered alternative hypotheses, we found a significant progressive decrease of the average gFA values (JTp=0.015), while the p-values for the JT-analysis on the global connectivity strength and average ADC values were not significant (JTp=0.48, JTp=0.14, respectively) (FigureS3).

Post-hoc ANCOVA revealed a significant impairment of the average ADC in stage-III patients compared to HCs (p=0.019). No other pair-wise differences of global network measures were found between the HC, stage-II, and stage-III groups.

When performing a nodal JT-analysis for ordered nodal connectivity-strength impairments {HC ≥ stage II ≥ stage III}, 7 out of 82 brain regions demonstrated a significant cross-group progressive decrease in nodal strength (JT-test, uncorrected p<0.05), namely right superior temporal cortex and insula, and left frontal pole, pericalcarine, lingual, fusiform and transverse temporal cortices. Two of these 7 regions were also identified as vulnerable regions in the main analyses on the complete dataset. None of the nodal JT-test survived multiple comparison correction.

Age, gender and handedness covariates were included in all the analyses.

*Relationship between connectivity strength and processing speed*

Similarly to the main finding, there was a significant positive association between the processing speed and the overall network strength when considering the 63 MAGNETOM-Trio HCs (r=0.29, p=0.023). The correlation coefficient between the processing speed and the overall network strength was positive for the other subject groups, but did not reach statistical significance (MAGNETOM-Trio EPPs (n=38): r=0.15, p=0.38; MAGNETOM-Prisma HCs (n=13): r=0.21, p=0.49; MAGNETOM-Prisma EPPs (n=19): r=0.43, p=0.064). In the MAGNETOM-Trio HCs, the association between processing speed and brain connectivity strength was driven by the strength of the connection between the identified 22 vulnerable regions (r=0.42, p=0.00072) (FigureS10). There was also a significant correlation between the MAGNETOM-Prisma EPPs processing speed and connectivity strength between the vulnerable regions (r=0.50, p=0.029) (FigureS10). These analyses on 4 distinct subgroups of the original dataset support the main finding that the connectivity strength between vulnerable brain regions selectively correlates with processing speed.

**7. Brain connectivity analyses restricted to right-handed subjects**

There was an unbalance in the proportion of right-handed subjects between the patients classified in stage II and stage III, and across the patients classified in stages IIIa, IIIb and IIIc. A categorical handedness covariate was added to all the analyses reported in the main manuscript. Moreover, in order to exclude a major influence of the subjects’ handedness on our results, when possible we repeated our analyses including right-handed subjects only. In particular: (i) we investigated the presence of progressive brain connectivity impairments from HCs to stage II patients to stage III patients, both the global network level and at the nodal level; (ii) we assessed the distribution of the patients in the sLDA feature space with respect to the subjects’ handedness.

In our complete dataset we had 66 right-handed HCs and 64 right-handed EPPs. The right-handed EPPs were subdivided into the clinical stages II (n=25) and III (n=39). Consistently with the main analyses, we found a significant ordered decrease {HC ≥ stage II ≥ stage III} in brain network connectivity strength (JTp=0.0042) and whole-brain tract-average gFA (JTp=0.047), and a ordered increase of whole-brain tract-average ADC (JT-p=0.020). Post-hoc ANCOVA revealed a significant impairment of the overall brain connectivity strength in stage-III patients compared to HCs (p=0.022, d=0.46). There was a trend of decreased average gFA (p=0.063, d=0.37) and increased average ADC (p=0.077, d=0.36) in stage-III patients compared to HCs. No significant pair-wise differences were found between stage-II patients and HCs or between stage-II and stage-III patients. Next, we performed a nodal JT-analysis for ordered nodal connectivity -strength impairments {HC ≥ stage II ≥ stage III} to identify the brain regions that contribute the most to the global effect. A total of 6 out of 82 brain regions demonstrate a significant cross-group progressive decrease in the nodal strength (JT-test, uncorrected p<0.05), namely: left pars opercularis and superior parietal cortices, right pars opercularis and lateral occipital cortices, right pallidum and right caudate. All these 6 regions belong to the vulnerable region set identified in the main analyses. No nodal test survived FDR-correction in this subjects’ subgroup.

Finally, we assessed the distribution of the patients in the sLDA feature space with respect to the subjects’ handedness. We did not find any spatial patterns in the distribution of the right-handed and left-handed subjects in the sLDA feature space, indicating that the subjects’ handedness is not a major driver of data classification (FigureS9).

**8. Network measure impairments in early psychosis patients**

As a preliminary analysis, we asked whether global brain connectivity alterations are already present (or detectable) in early psychosis patients (71 EPPs) with respect to age-, gender- and handedness-matched healthy controls (76 HCs). The following global connectivity-measure were considered: (i) overall network strength (NS) (i.e., total number of reconstructed streamlines (NOS) connecting pairs of cortical and subcortical regions); (ii) network efficiency (NE); (iii) average clustering coefficient (NC); (iv) whole-brain tract-average gFA; (v) whole-brain tract-average ADC. EPPs showed decreased NS (p=0.00086, d=0.55), NE (p=0.010, d=0.42) and NC (p=0.015, d=0.40) when compared to HCs (ANCOVA analysis, controlling for age, gender, handedness and scanner-upgrade; effect size was quantified with Cohen’s d coefficient) (FigureS2). However, the alterations of network efficiency and clustering coefficient were mainly driven by the NS effect, resulting in a non-significant EPPs-HCs NE or NC difference when controlling for NS (NE: p=0.14; NC: p=0.40) (FigureS4). We found a trend-level decrease of whole-brain tract-average gFA (p=0.051) and a trend-level increase of whole-brain tract-average ADC (p=0.068) (FigureS2).

Next, we investigated whether brain connectivity measures follow an increasing (or decreasing) pattern across the early psychosis clinical stages, by means of Jonckheere-Terpststra (JT) statistical testing for ordered alternative hypotheses {HC ≥ stage II ≥ stage III} (Figure2 in the main manuscript and FigureS5). We found a significant progressive decrease of NS (JTp=0.00027), NE (JTp =0.0027), NC (JTp =0.0042) and average gFA (p_JT_=0.038), with connectivity measures higher in HCs, intermediate in stage II patients and lower in stage III patients. The NE and NC effects were mainly driven by the NS effect. Tract-average ADC progressively increased across groups (JTp=0.0070).

Post-hoc multi-factor ANCOVA analyses revealed a significant impairment in the connectivity measures in the stage III patients compared to HCs (NS: p=0.00085, d=0.63; average gFA: p=0.038, d=0.39; average ADC: p=0.021, d=-0.42) (Figure2 in the main manuscript). There was a trend for decreased NS in stage II patients compared to HCs (p=0.074, d=0.41). No significant pair-wise differences were found between stage II patients and HCs for the other connectivity measures, or between stage II and stage III patients (FigureS5).

Considering that network density (i.e., the relative number of edges in a network) can have a significant impact on connectivity measures such as network strength, network efficiency and average clustering coefficient^29^, we tested for possible group-differences. No difference in network density was found between patients and controls (EPPs (average and standard deviation network density values): 0.23±0.01, HCs: 0.24±0.01; p=0.36), between stage II and stage III patients (II: 0.23±0.01, III: 0.24±0.01; p=0.24) or among stages IIIa-c patients (IIIa: 0.24±0.01, IIIb: 0.23±0.01, IIIc: 0.24±0.01; p=0.60).

**9. Brain network hubs**

Brain hubs are cortical or subcortical regions that occupy a central position in the overall brain-network topology. These regions play a fundamental role in maintaining proper communication and integration of information across the brain network, are associated with higher-order brain functions^30^, and are preferentially affected in a variety of psychiatric and neurological disorders^31^. Brain hubs and the subnetwork they form (i.e., the rich-club^32^) are implicated in schizophrenia pathophysiology and demonstrate impaired structural connectivity both in chronic schizophrenia patient^30,33^ and in subject with clinical or genetic risk for developing schizophrenia^34,35^.

In the present work, we followed a data-driven approach to identify vulnerable and progressively disrupted brain regions in early psychosis patients. To assess the overlap between those vulnerable regions and network hubs, we identified the network hubs as the 15% largest-degree nodes of the group-representative brain network. The group-representative brain network was obtained by considering the connections present in at least 50% of the subjects included in this study (patients and control subjects). The 13 brain regions that scored as network hubs were the following: bilateral superior parietal and superior frontal cortices, precunei, thalami, putamen and pallidum nuclei, and left rostral middle frontal cortex. This hubs’ set is consistent with previous reports^32^. 7 out of the 13 brain hubs also appeared as part of the vulnerable subnetwork (TableS3). We also note that, according to abundant literature^30,36,37^, the rich-club (a strongly inter-connected set of cortical hubs) includes the bilateral superior frontal, superior parietal, precunei and insula regions. 6 out of the 8 rich-club regions were also part of the vulnerable subnetwork detected in this study (TableS3).

**References**

1. Baumann PS, et al. Treatment and Early intervention in psychosis program (TIPP-Lausanne): Implementation of an early intervention program for psychosis in Switzerland. *Early Interv Psychiatry* 2013; **7**(3): 322-328.

2. Yung AR, et al. Mapping the onset of psychosis: The Comprehensive Assessment of at-risk-mental-states. *Aust N Z J Psychiatry* 2005; **39**(11-12): 964-971.

3. Hickie IB, et al. Applying clinical staging to young people who present for mental health care. *Early Interv Psychiatry* 2013; **7**(1): 31-43.

4. Alameda L, et al. Childhood sexual and physical abuse: Age at exposure modulates impact on outcome in early psychosis patients. *Physiological Medicine* 2015; **45**(13): 2727-2736.

5. McGorry PD, Nelson B, Goldstone S, Yung AR. Clinical staging: A heuristic and practical strategy for new research and better health and social outcome for psychotic and related mood disorders. *Can J Psychiatry* 2010; **55**(8): 486-497.

6. American Psychiatric Association (APA). Diagnostic and statistical manual of mental disorders. *Washington, DC: American Psychiatric Association* 2014; 4^th^ ed.

7. Kay SR, Fiszbein A, Opfer LA. The Positive And Negative Syndrome Scale (PANSS) for schizophrenia. *Schizophr Bull* 1987; **13**(2): 261-276.

8. Andreasen NC, et al. Antipsychotic dose equivalent and dose-years: A standardized method for comparing exposure to different drugs. *Biol Psy* 2010; **67**(3): 255-262.

9. Garden DM, et al. International consensus study of antipsychotic dosing. *Am J Psychiatry* 2010; **167**(6): 689-693.

10. Fischl B, Salat DH, Busa E, et al. Whole brain segmentation: Automated labeling of neuroanatomical structures in the human brain. *Neuron* 2002; **33**: 341-355.

11. Desikan RS, et al. An automated labeling system for subdividing the human cerebral cortex on MRI scans into gyral based regions of interest. *Neuroimage* 2006; **31**: 968-980.

12. Jenkinson M, Bannister P, Brady JM, Smith SM. Improved optimisation for the robust and accurate linear registration and motion correction of brain images. *NeuroImage* 2002; **17**(2): 825-841.

13. Yendiki A, et al. Spurious group difference due to head motion in a diffusion MRI study. *Neuroimage* 2014; **88**: 79-90.

14. Wedeen van J, et al. Mapping complex tissue architecture with diffusion spectrum magnetic resonance imaging. *Mag Reson Med* 2005; **54**(6): 1377-1386.

15. Tuch DS. Q-ball imaging. *Mag Reson Med* 2004; **52**(6): 1358-1372.

16. Daducci A, et al. The Connectome Mapper: An open-source processing pipeline to map connectomes with MRI. *PloS One* 2012; **7**(12): e48121.

17. Zalesky A, et al. Connectome sensitivity or specificity: Which is more important? *Neuroimage* 2016; **142**: 407-420.

18. de Reus MA, van den Heuvel M. Estimating false positives and negatives in brain networks. *Neuroimage* 2013; **70**: 402-409.

19. Rubinov M, Sporns O. Complex network measures of brain connectivity: Uses and interpretations. *Neuroimage* 2010; **52**(3): 1059-1069.

20. Onnela J-P, Saramaki J, Kertesz J, Kaski K. Intensity and coherence of motifs in weighted complex networks. *Phys Rev E* 2005; **71**: 056103.

21. Bullmore E, Sporns O. The economy of brain network organization. *Nat Rev Neurosci* 2012; **13**: 336-349.

22. Sporns O. Network attributes of segregation and integration in the human brain. *Curr Opin Neurobiol* 2013; **23**(3): 162-171.

23. Kern RS, et al. The MATRICS consensus cognitive battery, part 2: Co-norming and standardization. *Am J Psychiatry* 2008; **165**(2): 214-220.

24. Nuechterlein KH, et al. The MATRICS consensus cognitive battery, part 1: Test selection, reliability, and validity. *Am J Psychiatry* 2008; **165**(2): 203-213.

25. James G, Witten D, Hastie T, Tibshirani R. An introduction to statistical learning. *Springer Texts in Statistics* 2013; ISBN 978-1-4616-7137-0.

26. Mcgorry PD, et al. Biomarkers and clinical staging in psychiatry. *World Psychiatry* 2014; **3**: 211-223.

27. Kochunov P, et al. Association of white matter with core cognitive deficits in patients with schizophrenia. *JAMA Psychiatry* 2017; **74**(9):958-966.

28. Clemmens L, Hastie T, Witten D, Ersboll B. Sparse Discriminant Analysis. *Technometrics* 2011; **53**(4): 406-413.

29. Meskaldji DE, et al. Comparing connectomes across subjects and populations at different scales. *Neuroimage* 2013; **80**: 416-425.

30. van den Heuvel MP, et al. Abnormal rich club organization and functional brain dynamics in schizophrenia. *JAMA Psychiatry* 2013; **70**(8): 783-792.

31. Crossley NA, et al. The hubs of the human connectome are generally implicated in the anatomy of brain disorders. *Brain* 2014; **137**(8): 2382-2395.

32. van den Heuvel MP, Sporns O. Rich-club organization of the human connectome. *J Neurosci* 2011; **31**(44): 15775-15786.

33. Klauser P, et al. White matter disruptions in schizophrenia are spatially widespread and topologically converge on brain network hubs. *Schizophr Bull* 2017; 43(2): 425-435.

34. Schmidt A, et al. Structural network disorganization in subjects at clinical high risk for psychosis. *Schizophr Bull* 2017; **43**(3): 583-591.

35. Collin G, et al. Affected anatomical rich club and structural-functional coupling in young offspring of schizophrenia and bipolar disorder patients. *Biol Psychiatry* 2017; **82**: 746-755.

36. Collin G, et al. Impaired rich club connectivity in unaffected siblings of schizophrenia patients. *Schizophr Bul* 2014; **40**(2): 438-448.

37. Ball G, et al. Rich-club connectome organization of the newborn human brain. *Proc Natl Acad Sci USA* 2014; **111**(20): 7456-7461.

**Figure S1 – sLDA training and LOOCV errors**

sLDA training error (A) and LOOCV error (B) for the range of λ-values [10, 45]. The value of the sLDA parameter λ corresponds to the number of non-zero feature-loading in each resulting LDD. B: the continuous line represents the chance-level LOOCV-error for 4-group classification; the dotted line represents the naïve classifier LOOCV-error. By visually inspecting the two plots, we can observe that the training error monotonically increases with increasing λ (i.e., with increasingly flexible classification models). On the other side, the LOOCV error is high for low λ-values, then tends to decrease reaching below-chance error values, and finally increase for high λ-values indicating overfitting. The minimum average LOOCV classification error was achieved for λ = 38.


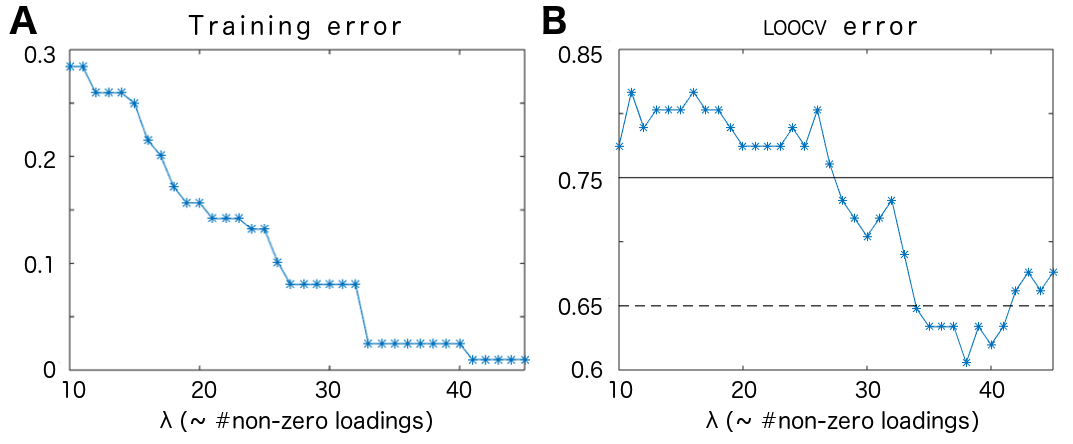


**Figure S2 – EPP-HC group-comparison**

Brain connectivity measures for healthy controls (HC) and early psychosis patients (EPP). NS: network strength; NE: network efficiency; NC: network clustering coefficient: whole-brain tract-average gFA; whole-brain tract-average ADC. Residuals after correction for age, gender, handedness and scanner-upgrade are shown. EPP-HC ANCOVA p-value and Cohen’s d coefficient are reported for each connectivity measure.


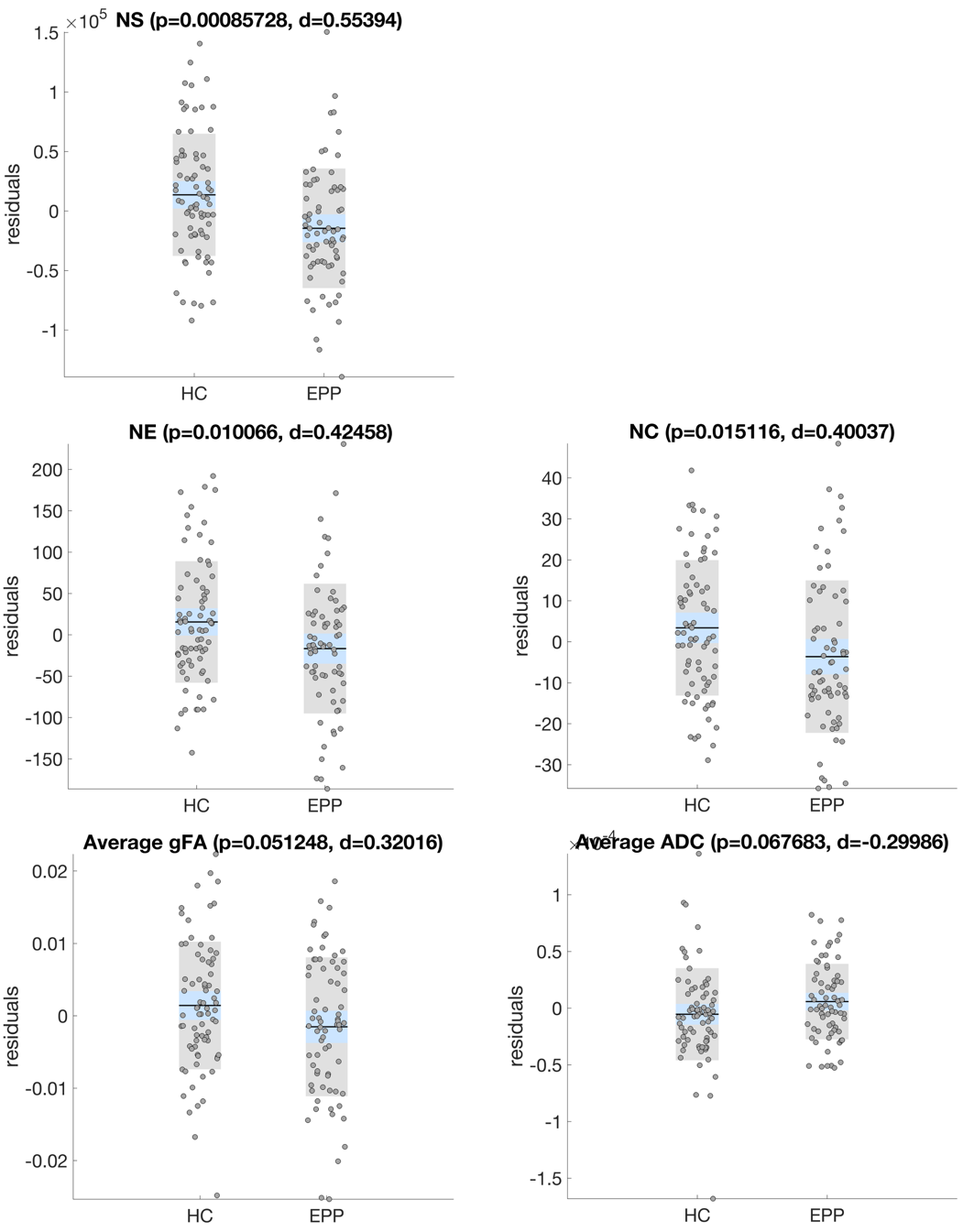


**Figure S3 – HC, stage-II and stage-III group-comparison for Trio and Prisma cohorts**

Scatter-plots of overall brain connectivity strength and whole-brain tract-average gFA and ADC values for healthy controls (HC), stage II and stage III patients who were scanned on the Trio system (first row) or on the Prisma system (second row). Residuals after correction for age, gender and handedness are reported. The size of each group is reported in the first two plots (first column). For single-group scatter-plots, the standard error of the mean (light blue area) and the group standard deviation (grey area) around the group mean (black line) are reported. Blue lines with asterisk represent statistically significant group-differences (uncorrected p<0.05); grey lines represent trend-level differences (uncorrected p<0.1). JT p-values for ordered alternative hypotheses testing ({HC ≥ stage II ≥ stage III} for connectivity strength and gFA, {HC ≤ stage II ≤ stage III} for ADC) are reported; asterisks indicate statistically significant JT test (uncorrected p<0.05).


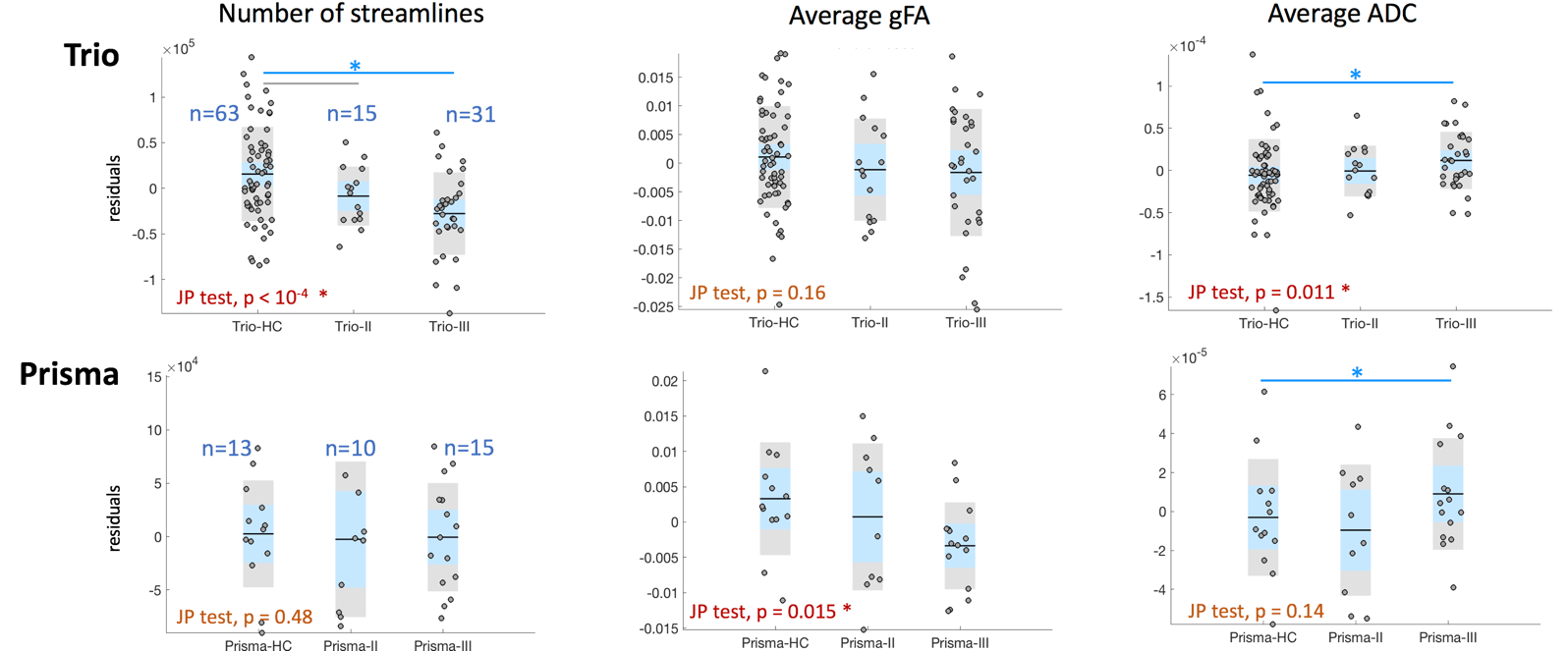


**Figure S4 – EPP-HC network efficiency and average clustering coefficient**

Network efficiency (NE) and average clustering coefficient (NC) values for healthy controls (HC) and early psychosis patients (EPP) after correcting for the overall network strength (NS). Residuals after correction for NS, age, gender, handedness and scanner-upgrade are shown. EPP-HC ANCOVA p-values are reported for both connectivity measures.


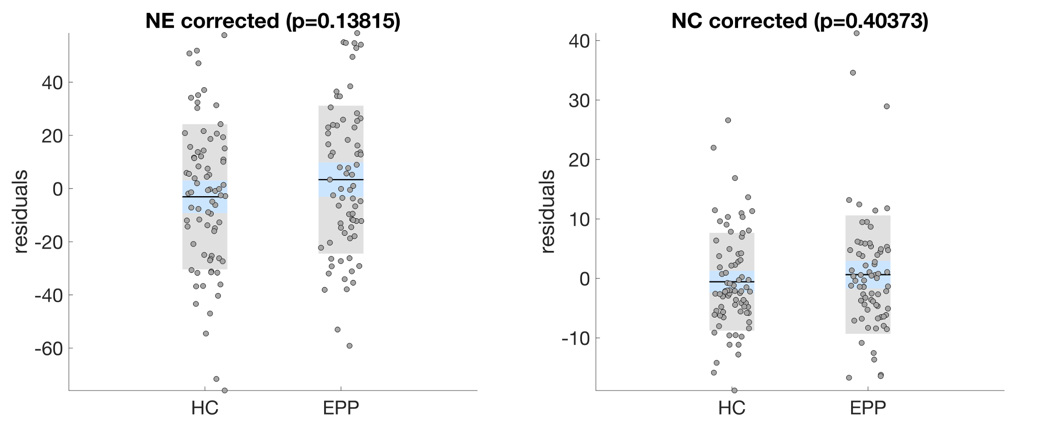


**Figure S5 – Progression impairment of network efficiency and average clustering coefficient values**

Scatter-plots of brain network efficiency and average clustering coefficient for HCs, stage II and stage III patients. Residuals after correction for age, gender, handedness and scanner-upgrade (A), and after correction for age, gender, handedness, scanner-upgrade and overall network strength (B) are reported. Grey dotted lines indicate average values for the Hcs group. P-values of Jonckheere-Terpstra tests for ordered alternative hypotheses {HC ≥ stage II ≥ stage III} are reported.


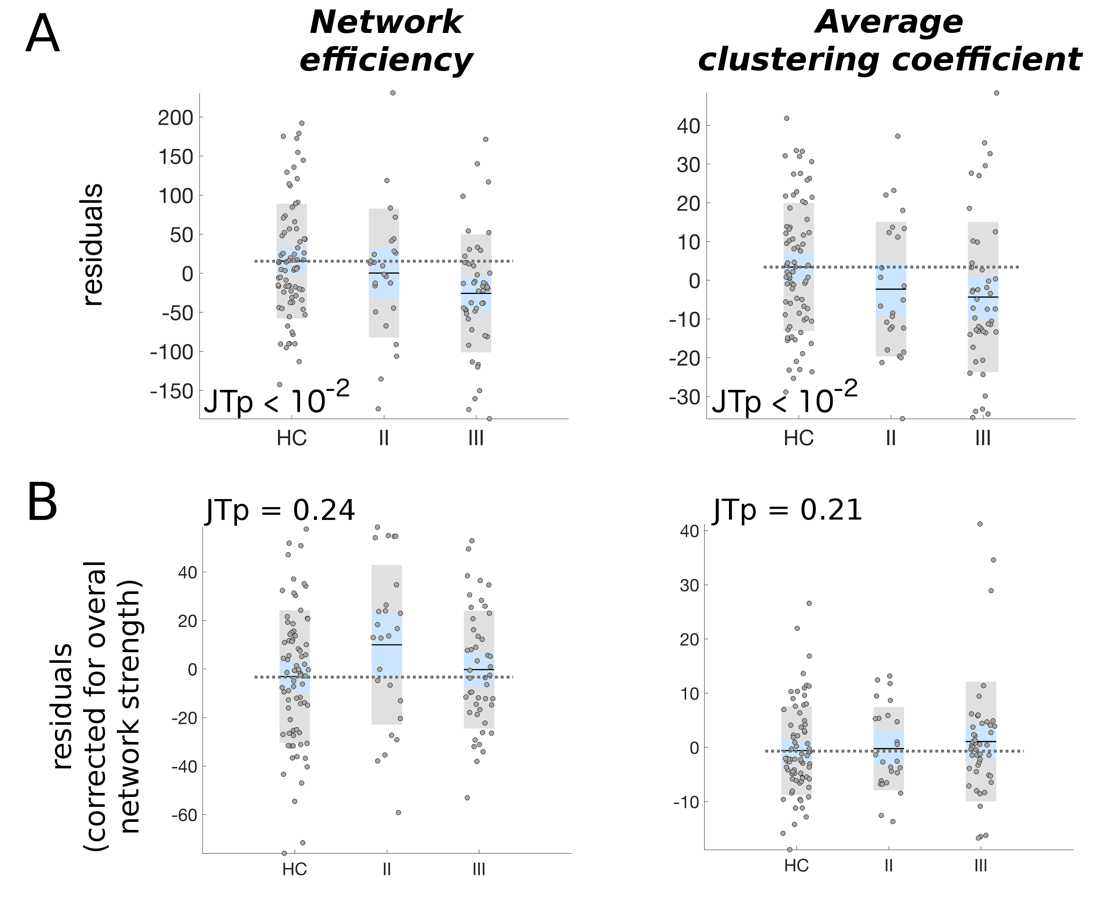


**Figure S6 – Vulnerable brain regions identified on the complete and Trio datasets**

Cortical surface plot of nodal JT p-values for ordered impairment of nodal strength values {HC ≥ stage II ≥ stage III} (uncorrected p<0.05; cortical regions with uncorrected p≥0.05 are colored in grey) from whole dataset analysis (Trio and Prisma data, left column -replicating Figure3A-) and from the Trio data analysis (right column). The lists of regions with JT-p<0.05 for the two analyses are also reported (** indicates regions surviving FDR-correction).


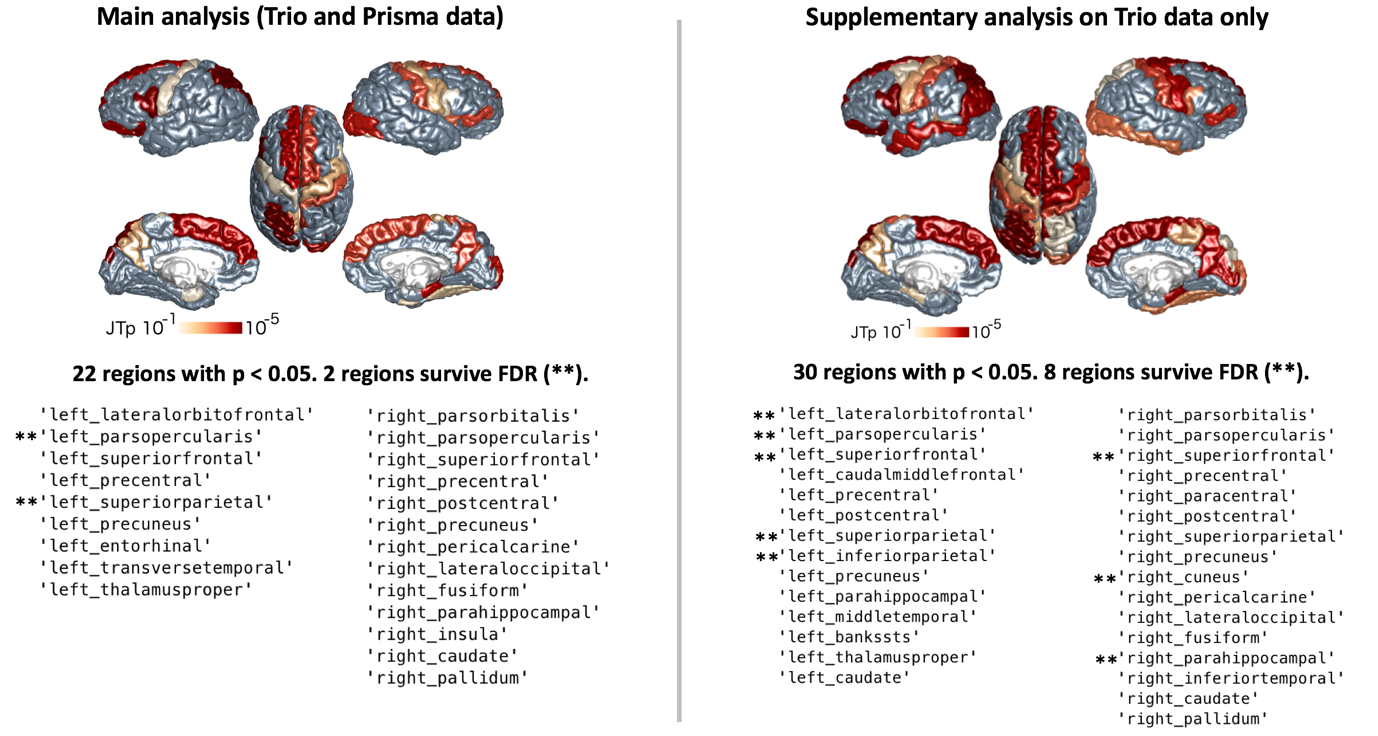


**Figure S7 – Linear discriminant directions’ loadings**

Cortical-surface plots of the nodal loadings (i.e., coefficients of the linear discriminative functions) defining the three linear discriminant directions (LDDs) obtained from sLDA analysis, for λ=38. Loadings’ absolute values are represented, with darker tones highlighting the brain regions that mostly contribute to the inter-class discrimination, along a given LDD. Grey areas correspond to LDD-loadings equal to zero (i.e., regions non-contributing to the inter-class discrimination, along a given LDD).


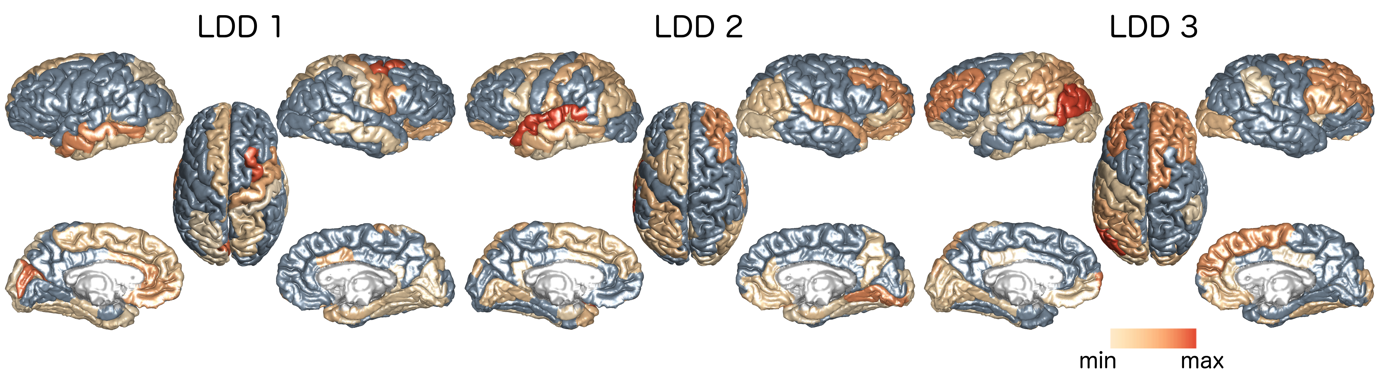


**Figure 8 – sLDA feature space and scanner upgrade**

Patients’ representation in the sLDA feature space when sLDA is performed on all the available EPPs (including both MAGNETOM-Trio and MAGNETOM-Prisma data). Each dot represents a single patient. First row: data projection on the plane defined by the first and second linear discriminant directions (LDDs); second row: data projection on the plane defined by the first and third LDDs. Left column: subjects are color-coded according to their clinical staging condition (replication of Figure4 in the main manuscript); right column: subjects are color-coded in blue if imaged before the scanner upgrade (MAGNETOM-Trio system), or in green if imaged after the scanner upgrade (MAGNETOM-Prisma system). There is no evident pattern of data distribution in the sLDA feature space with respect to the scanner upgrade variable.


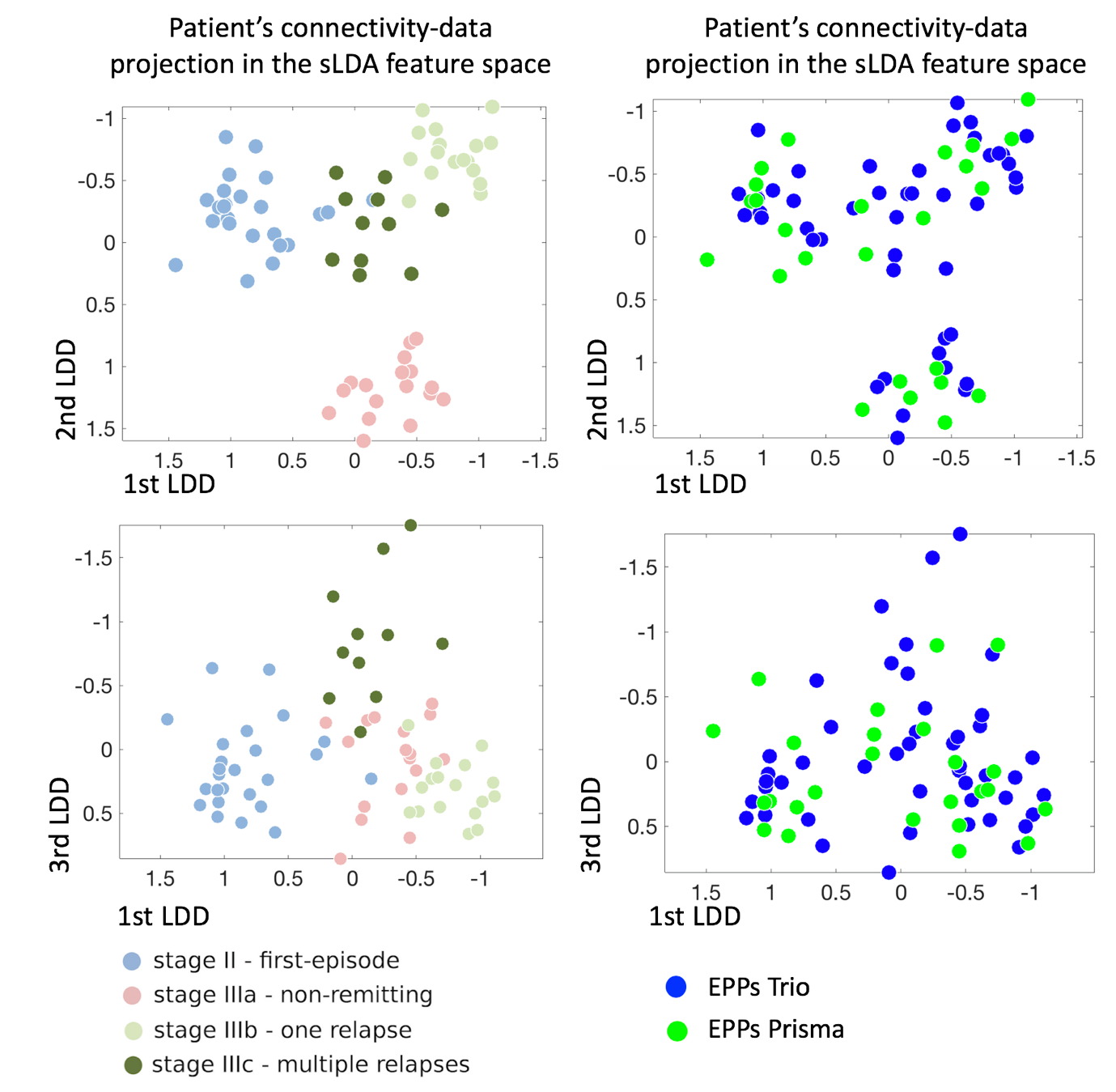


**Figure 9 – sLDA feature space and subjects’ handedness**

Patients’ representation in the sLDA feature space when sLDA is performed on all the available EPPs. Each dot represents a single patient. First row: data projection on the plane defined by the first and second linear discriminant directions (LDDs); second row: data projection on the plane defined by the first and third LDDs. Left column: subjects are color-coded according to their clinical staging condition (replication of Figure4 in the main manuscript); right column: subjects are color-coded in red if right-handed, or in black if left-handed. There is no evident pattern of data distribution in the sLDA feature space with respect to the handedness variable.


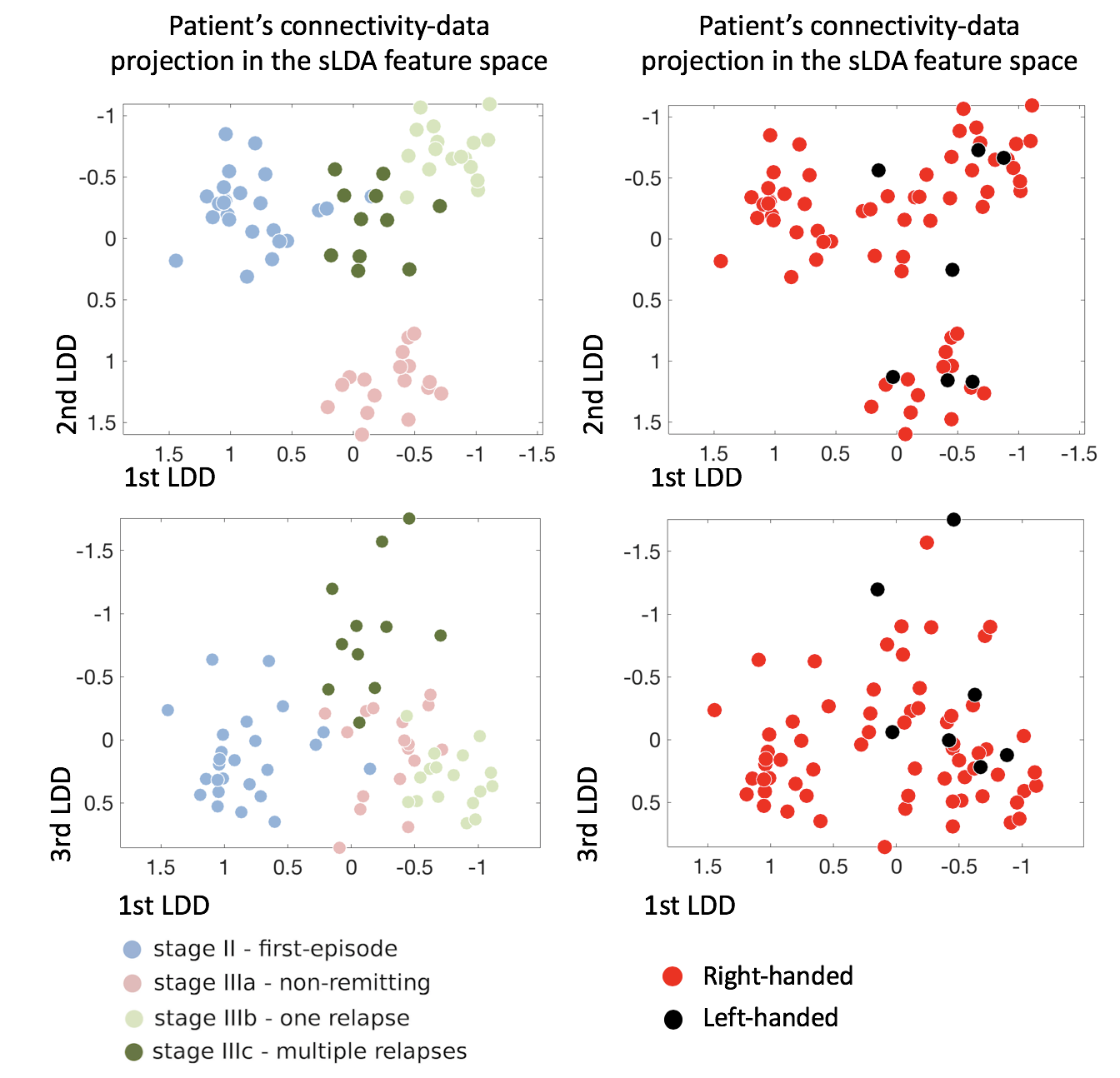


**Figure S10 – Connectivity strength and processing speed in Trio and Prisma datasets**

Relationship between the brain connectivity strength between the 22 vulnerable grey matter regions (left side of the figure) or between the other brain regions (right side of the figure), and the processing speed in HCs (blue dots) and EPPs (red dots) imaged before (MAGNETOM-Trio, first row) or after (MAGNETOM-Prisma, second row) the scanner upgrade. Pearson’s correlation coefficients and relative p-values are reported at the top of each scatter plot; the asterisks indicate statistically significant correlations (uncorrected p < 0.05). Grey lines represent least-square linear fits.


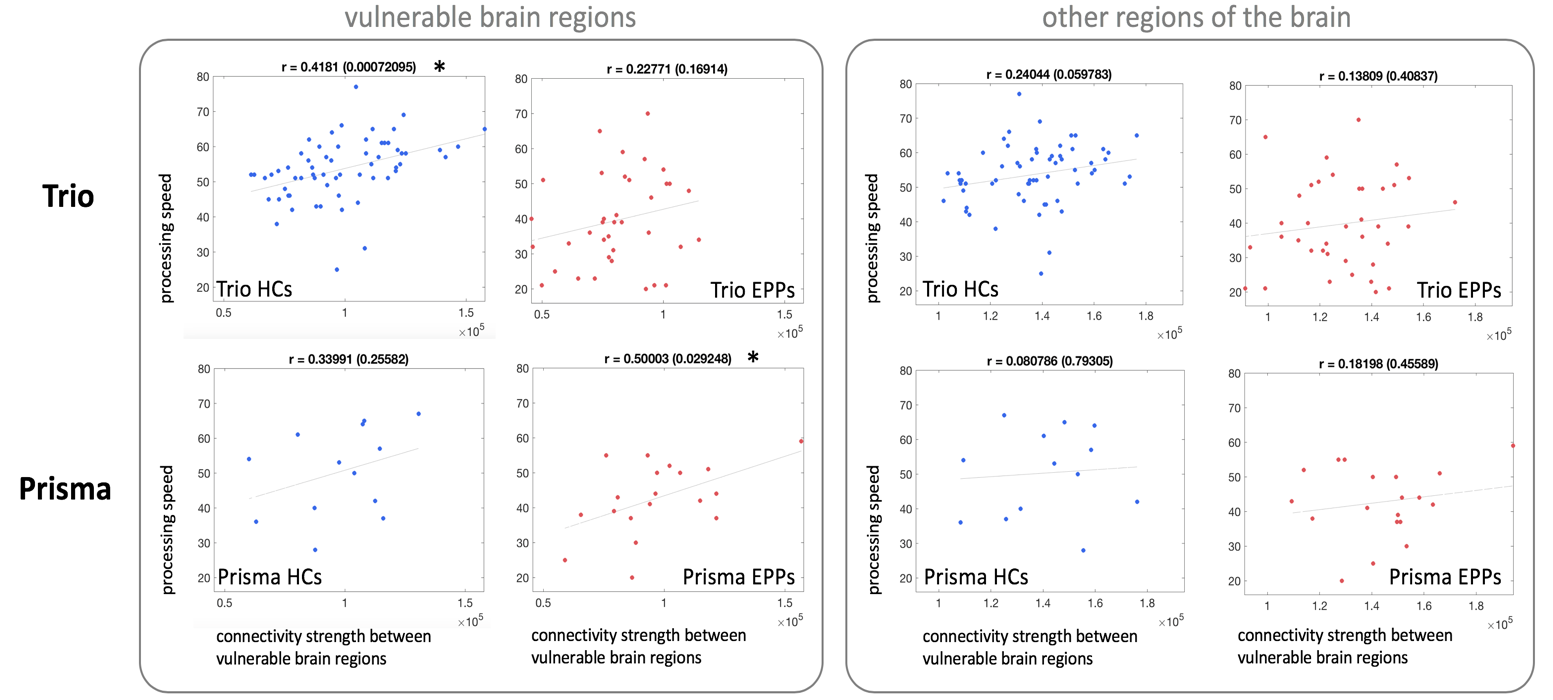


**Table S1 – Trio and Prisma cohorts**

Sample size for subjects imaged before (MAGNETOM-Trio) or after (MAGNETOM-Prisma) the scanner upgrade.

| Subject groups | TRIO | PRISMA | TOTAL |
| --- | --- | --- | --- |
|  |  |  |  |
| HC | 63 | 13 | 76 |
| EPP – stage II | 15 | 10 | 25 |
| EPP – stage III | 31 | 15 | 46 |
| EPP – stage IIIa | 10 | 7 | 17 |
| EPP – stage IIIb | 12 | 5 | 17 |
| EPP – stage IIIc | 9 | 3 | 12 |

**Table S2 – Summary of group-differences for Trio and Prisma cohorts**

Cohen’s d coefficient for group-comparisons of different connectivity measures, and between different subject groups. The third and fourth columns contain d-values obtained when including in the analyses only subjects scanned on the Trio or on the Prisma system, while the last column contain d-values from the main analyses on Trio and Prisma data (including a ‘scanner upgrade’ covariate). The Cohen’s d coefficient is defined as the difference between the two groups’ mean values, normalized by the pooled standard deviation, so that a negative d-value indicates that the first group (e.g., EPP) has a lower mean than the second group (e.g., HC) reported in the column ‘groups’. For each row (i.e., for each connectivity measure and group-comparison), d-values with the same sign indicate coherent mean-differences across datasets. ^#^ p-value < 0.1; * p-vale < 0.05; ** p-value < 0.01.

| Connectivity measures | groups | TRIO | PRISMA | TOTAL |
| --- | --- | --- | --- | --- |
|  |  |  |  |  |
| Overall connectivity strength | EPP-HC | -0.77** | -0.07 | -0.47** |
| Whole-brain tract-average gFA | EPP-HC | -0.27 | -0.62^#^ | -0.23 |
| Whole-brain tract-average ADC | EPP-HC | +0.45^#^ | +0.15 | +0.30^#^ |
|  |  |  |  |  |
| Overall connectivity strength | III-HC | -0.87** | -0.05 | -0.54** |
| Whole-brain tract-average gFA | III-HC | -0.28 | -0.93* | -0.32^#^ |
| Whole-brain tract-average ADC | III-HC | +0.45* | +0.40 | +0.42* |
|  |  |  |  |  |
| Overall connectivity strength | II-HC | -0.51^#^ | -0.27 | -0.38^#^ |
| Whole-brain tract-average gFA | II-HC | -0.31 | -0.34 | -0.28 |
| Whole-brain tract-average ADC | II-HC | +0.19 | +0.07 | +0.10 |
|  |  |  |  |  |
| Overall connectivity strength | III-II | -0.42 | -0.12 | -0.15 |
| Whole-brain tract-average gFA | III-II | +0.0015 | -0.56 | -0.16 |
| Whole-brain tract-average ADC | III-II | +0.37 | +0.88* | +0.42^#^ |

**Table S3 – Vulnerable brain regions**

List of brain regions with p-value below uncorrected significant threshold alpha=0.05 (nodal JT-test for HC ≥ stage II ≥ stage III ordered alternative hypotheses). The ‘*’ symbol indicates regions surviving multiple comparison correction (FDR<0.05); the ‘#’ symbol indicates hub regions; the ‘$’ symbol indicates rich-club regions.

| Brain region | p-value |
| --- | --- |
|  |  |
| Left Superior parietal *** #$** | **0.0002** |
| Left Pars opercularis ***** | **0.0008** |
| Left Lateral orbito-frontal | 0.0051 |
| Left Superior frontal **#$** | 0.0069 |
| Right Parahippocampal | 0.0076 |
| Right Caudate | 0.0089 |
| Right Lateral-occipital | 0.0104 |
| Right Precuneus **#$** | 0.0135 |
| Right Pars orbitalis | 0.0149 |
| Right Superior frontal **#$** | 0.0146 |
| Right Postcentral | 0.0171 |
| Left Thalamus proper **#** | 0.0205 |
| Right Insula **$** | 0.0210 |
| Left Precuneus **#$** | 0.0336 |
| Right Precentral | 0.0342 |
| Left Transverse temporal | 0.0365 |
| Right Fusiform | 0.0401 |
| Right Pallidum **#** | 0.0418 |
| Right Pericalcarine | 0.0434 |
| Right Pars opercularis | 0.0452 |
| Left Precentral | 0.0452 |
| Left Entorhinal | 0.0470 |
